# Asymmetric distributed trust

**DOI:** 10.1007/s00446-024-00469-1

**Published:** 2024-05-28

**Authors:** Orestis Alpos, Christian Cachin, Björn Tackmann, Luca Zanolini

**Affiliations:** 1 Common Prefix, Bern, Switzerland; 2 University of Bern, Bern, Switzerland; 3 Institute of Computer Science, University of Bern, Bern, Switzerland; 4 DFINITY Foundation, Zürich, Switzerland

## Abstract

Quorum systems are a key abstraction in distributed fault-tolerant computing for capturing trust assumptions. They can be found at the core of many algorithms for implementing reliable broadcasts, shared memory, consensus and other problems. This paper introduces *asymmetric Byzantine quorum systems* that model subjective trust. Every process is free to choose which combinations of other processes it trusts and which ones it considers faulty. Asymmetric quorum systems strictly generalize standard Byzantine quorum systems, which have only one global trust assumption for all processes. This work also presents protocols that implement abstractions of shared memory, broadcast primitives, and a consensus protocol among processes prone to Byzantine faults and asymmetric trust. The model and protocols pave the way for realizing more elaborate algorithms with asymmetric trust.

## Introduction

Byzantine quorum systems [1] are a fundamental primitive for building resilient distributed systems from untrusted components. Given a set of nodes, a quorum system captures a trust assumption on the nodes in terms of potentially malicious protocol participants and colluding groups of nodes. Based on quorum systems, many well-known algorithms for *reliable broadcast*, *shared memory*, *consensus* and more have been implemented; these are the main abstractions to synchronize the correct nodes with each other and to achieve consistency despite the actions of the faulty, so-called *Byzantine* nodes.

Traditionally, trust in a Byzantine quorum system for a set of processes $\mathcal {P}$ has been *symmetric*. In other words, a global assumption specifies which processes may fail, such as the simple and prominent *threshold quorum* assumption, in which any subset of $\mathcal {P}$ of a given maximum size may collude and act against the protocol. The most basic threshold Byzantine quorum system, for example, allows all subsets of up to $f < n/3$ processes to fail. Some classic works also model arbitrary, non-threshold symmetric quorum systems [1, 2], but it is unknown if these have been used in practice.

However, trust is inherently subjective. *De gustibus non est disputandum—There is no disputing about taste.* Estimating which processes will function correctly and which ones will misbehave may depend on personal taste. A myriad of local choices influences one process’ trust in others, especially because there are so many forms of “malicious” behavior. Some processes might not even be aware of all others, yet a process should not depend on unknown third parties in a distributed collaboration. How can one model asymmetric trust in distributed protocols? Can traditional Byzantine quorum systems be extended to subjective failure assumptions? How do the standard protocols generalize to this model?

*Asymmetric trust* In this paper, we answer these questions and introduce models and protocols for asymmetric distributed trust. We formalize *asymmetric (Byzantine) quorum systems* for asynchronous protocols, in which every process can make its own assumptions about Byzantine faults of others. We introduce several protocols with asymmetric trust that strictly generalize the existing algorithms, which require common trust.

Our formalization takes up earlier work by Damgård et al. [3] and starts out with the notion of a fail-prone system that forms the basis of a symmetric Byzantine quorum system. A global fail-prone system for a process set $\mathcal {P}$ contains all maximal subsets of $\mathcal {P}$ that might jointly fail during an execution. In an asymmetric quorum system, every process specifies its *own* fail-prone system and a corresponding set of local quorums. These local quorum systems satisfy a *consistency condition* that ranges across all processes and a local *availability condition*, and generalize symmetric Byzantine quorum system according to Malkhi and Reiter [1].

*Protocols with asymmetric quorums* Quorum systems are used within various fault-tolerant distributed protocols, here specifically within protocols for systems subject to Byzantine faults. An important aspect of our notion concerns its relation to existing protocols: it should be easy to generalize the known protocols to the asymmetric model, ideally simply by replacing the symmetric quorums with their asymmetric counterparts. Indeed this is the case for many, but not for all protocols described here. A different, generalized analysis is necessary in any case.

We show first that two existing protocols for emulating a shared regular register also work in the asymmetric model. Second, we introduce asymmetric Byzantine consistent and reliable broadcast primitives, for which we again only change the quorums compared to the protocols with symmetric quorums. Third, we address consensus, one of the most important primitives in distributed computing, and extend a randomized binary consensus protocol for asynchronous networks to work with asymmetric trust. The protocol relies on a common coin abstraction, for which a different implementation is needed.

Our randomized consensus takes up the award-winning, randomized, and signature-free implementation of consensus by Mostéfaoui et al. [4]. In its 2014 version, however, this protocol suffered from a liveness issue, which was corrected subsequently [5], although the fix added considerable complexity. The corrected algorithm offers the same asymptotic complexity in message and time as the original algorithm, but it requires more communication steps.

Through our randomized asymmetric consensus, we also introduce a novel way of fixing the problem in the original protocol. It retains the latter protocol’s simplicity, which is an appealing property. Obviously, our asymmetric consensus protocol can also be instantiated with symmetric threshold quorums to work in the same model as the protocol of Mostéfaoui et al. [4]. In order to clearly demonstrate the liveness issue and to show how our approach avoids it, we also include in this work a discussion of this randomized consensus algorithm in the symmetric-trust model.

In the traditional models for quorum-based systems, all correct processes uniformly benefit from the guarantees of a protocol as long as the initial assumption expressed by the fail-prone system holds. With subjective trust, this symmetry no longer exists. Some of the correct processes may have made assumptions that proved appropriate in an execution with actually faulty processes $F \subset \mathcal {P}$; we call these processes *wise*. Other correct processes, however, may have assumed that only a proper subset of *F* actually fails; these processes are *naïve* and they do not enjoy the same guarantees as the wise ones, even though they are correct. In particular, our protocols typically ensure safety only for wise processes and liveness depends on the existence of a sufficiently large group of wise processes.

*Motivation* Interest in consensus protocols based on Byzantine quorum systems has surged recently because of their application to permissioned blockchain networks [6, 7]. Typically run by a consortium, such distributed ledgers often use *Byzantine-fault tolerant (BFT)* protocols like PBFT [8], Tendermint [9], or HotStuff [10] for consensus that rely on symmetric threshold quorum systems. The Bitcoin blockchain and many other cryptocurrencies, which triggered this development, started from different assumptions and use so-called permissionless protocols, in which everyone may participate. Those algorithms capture the relative influence of the participants on consensus decisions by an external factor, such as invested “work” or “stake” in the system.

A middle ground between permissionless blockchains and BFT-based ones has been introduced by the blockchain networks of Ripple (https://ripple.com) and Stellar (https://stellar.org). Their stated model for achieving network-level consensus uses subjective trust in the sense that each process declares a local list of processes that it “trusts” in the protocol.

Consensus in the *Ripple* blockchain (and for the *XRP* cryptocurrency on the *XRP Ledger*) is executed by its validator nodes. Each node declares a *Unique Node List (UNL)*, which are validators that this node trusts, in the sense that “the given participant believes [they] will not conspire to defraud [the node].” At least up to around 2020, however, nodes have not really been free in their trust choice since “Ripple provides a default and recommended list which we [Ripple] expand based on watching the history of validators operated by Ripple and third parties” [11]. As of 2023, the XRP ledger documentation states that “currently the XRP Ledger Foundation and Ripple are known to publish recommended default lists of high quality validators ...” [12]. It is clear that two nodes that transact via the XRP ledger need to have some validators that they trust in common. But many questions are left open about the kind of decentralization offered by the Ripple protocol.

*Stellar* was created as an evolution of Ripple that shares much of the same design philosophy. The Stellar consensus protocol [13] powers the *Stellar Lumen (XLM)* cryptocurrency and introduces *federated Byzantine quorum systems (FBQS)*; they also capture subjective trust assumptions, but differ technically from asymmetric quorum systems. Stellar’s consensus protocol uses *quorum slices*, which are “the subset of a quorum that can convince one particular node of agreement.” In an FBQS, “each node chooses its own quorum slices” and “the system-wide quorums result from these decisions by individual nodes” [14].

*Contribution* The main motivation for this work is to understand how existing ideas of subjective trust, as manifested in the Ripple and Stellar blockchains, relate to traditional quorum systems. The formalization of asymmetric quorums provides a sound foundation for protocols with asymmetric trust. The protocols described here generalize well-known, classic algorithms in the literature and therefore look similar. This should be seen as a feature, actually, because simplicity and modularity are important guiding principles in science.

Our contributions are as follows:

- We introduce asymmetric Byzantine quorum systems formally in Sect. 4 as an extension of standard Byzantine quorum systems and discuss some of their properties.
- In Sect. 5, we show two implementations of a shared register, with single-writer, multi-reader regular semantics, using asymmetric Byzantine quorum systems.
- We examine broadcast primitives in the Byzantine model with asymmetric trust in Sect. 6. In particular, we define and implement Byzantine consistent and reliable broadcast protocols.
- In Sect. 7, we present the first asynchronous Byzantine consensus protocol with asymmetric trust. It uses randomization, provided by an asymmetric common coin protocol, to circumvent the impossibility of asynchronous consensus.

Before presenting the technical contributions, we discuss related work in Sect. 2 and state our system model in Sect. 3. A detailed discussion of the liveness issue in the existing signature-free Byzantine consensus protocol [4] and of our approach to fixing it appears in Appendix A.

## Related work

*Practical systems: Ripple and Stellar* The *Ripple* consensus protocol is run by an open set of validator nodes. The protocol uses votes, similar to standard consensus protocols, whereby each validator only communicates with the validators in its UNL. Each validator chooses its own UNL, which makes it possible for anyone to participate, in principle, similar to proof-of-work blockchains. Early investigations suggested that the intersection of the UNLs of every two validators should be at least 20% of each list [15], assuming that also less than one fifth of the validators in the UNL of every node might be faulty. An independent analysis by Armknecht et al. [16] later argued that this bound must be more than 40%. A technical report of Chase and MacBrough [17, Thm. 8] concludes, under the same assumption of $f < n/5$ faulty nodes in every UNL of size *n*, that the UNL overlap should actually be at least 90%.

However, the same paper also derives a counterexample to the liveness of the Ripple consensus protocol [17, Sect. 4.2] as soon as two validators don’t have “99% UNL overlap.” By generalizing the example, this essentially means that the protocol can get stuck *unless all nodes have the same UNL*. According to the standards of the field of distributed systems, though, a protocol needs to satisfy safety *and* liveness because achieving only one of these properties is trivial. Amores-Sesar et al. [18] confirm the prior analysis and exhibit a wider set of examples how safety and liveness may be violated in executions of the Ripple consensus protocol. They first show that the network may fork, even under the standard condition stated by Ripple on the overlap of UNLs, and then that the consensus protocol may lose liveness in the presence of only one Byzantine process, even if all the processes have the same UNL. These works, however, exploit arbitrary message delays, i.e., a period of asynchronous network behavior, which is not assumed by Ripple and arguably also unlikely to occur in practice.

The *Stellar consensus protocol (SCP)* also features open membership and lets every node express its own set of trusted nodes [13, 19]. Generalizing from Ripple’s flat lists of unique nodes, every node declares a collection of trusted sets called *quorum slices*, whereby a slice is “the subset of a quorum convincing one particular node of agreement.” A *quorum* in Stellar is a set of nodes “sufficient to reach agreement,” defined as a set of nodes that contains one slice for each member node. The quorum choices of all nodes together yield a *federated Byzantine quorum systems (FBQS)*. The literature on Stellar gives properties for FBQS and contains protocols that build on them, which have been implemented in the Stellar blockchain [19]. However, standard Byzantine quorum systems and FBQS are *not* comparable because (1) an FBQS when instantiated with the same trust assumption for all processes does not reduce to a symmetric quorum system and (2) existing protocols do not directly generalize to FBQS.

*Models of asymmetric trust* Starting from Stellar’s notions, García-Pérez and Gotsman [20] build a link from FBQS to existing quorum-system concepts by investigating a Byzantine reliable broadcast abstraction in an FBQS. They show that the *federated voting protocol* of Stellar [13] is similar to Bracha’s reliable broadcast [21] and that it implements a variation of Byzantine reliable broadcast on an FBQS for executions that contain, additionally, a set of so-called intact nodes. Losa et al. [22] have later formulated an abstraction of the consensus mechanism in the Stellar network by introducing *Personal Byzantine quorum systems* (PBQS). In contrast to the other notions of “quorums”, their definition does not require a global intersection among quorums. This may lead to several separate *consensus clusters* such that each one satisfies agreement and liveness on its own.

The FBQS and PBQS concepts, however, differ from the notion of a Byzantine quorum system in the literature. In particular, the characterization of their properties seems to take into account knowledge of which nodes are Byzantine, and their effects are therefore analyzed in the context of particular executions. Existing notions of symmetric quorum systems in the literature [1, 2] start from an a-priori assumption about all potentially faulty sets of nodes, through a fail-prone system [1]. This permits to study protocol-independent aspects of quorum systems.

Another approach for designing Byzantine fault-tolerant (BFT) consensus protocols has been introduced by Malkhi et al. [23], namely *Flexible BFT*. This notion guarantees higher resilience by introducing a new *alive-but-corrupt* fault type, which denotes processes that attack safety but not liveness. Malkhi et al. [23] also define *flexible Byzantine quorums* that allow processes in the system to have different faults models.

Our work, in contrast, goes back to the model of Damgård et al. [3]. It already contains the basic formulation of asymmetric trust and expresses it in the context of synchronous protocols for secure distributed computation with process-specific fail-prone systems. The model features only a consistency property, but omits liveness. Damgård et al. [3] also state a characterization of when an asymmetric Byzantine quorum system exists (with the so-called $B^3$), but give no proof. Their work has remained without impact until research on cryptocurrencies has revived interest in heterogeneous and subjective trust models.

*Signature-free randomized consensus* Mostéfaoui et al. [4] present a randomized, signature-free, and round-based asynchronous consensus algorithm for binary values. It achieves optimal resilience and takes $O(n^2)$ constant-sized messages. Randomization is achieved through a common coin as defined by Rabin [24]. Their binary consensus algorithm has been taken up for constructing the “Honey Badger BFT” protocol by Miller et al. [25], for instance. One important contribution of Mostéfaoui et al. [4] is a new binary validated broadcast primitive with a non-deterministic termination property; it has also found applications in other protocols [26].

Tholoniat and Gramoli [27] observe a liveness issue in the protocol by Mostéfaoui et al. [4] in which an adversary is able to prevent progress among the correct processes by controlling messages between them and by sending them values in a specific order.

In a later work, Mostéfaoui et al. [5] present a different version of their randomized consensus algorithm that does not suffer from the liveness problem anymore. The resulting algorithm offers the same asymptotic complexity in message and time as their previous algorithm [4], but requires more communication steps.

## System model

*Processes* We consider a system of *n* *processes* $\mathcal {P} = \{p_1, \dots , p_n\}$ that communicate with each other. The processes interact asynchronously with each other through exchanging messages. The system itself is asynchronous, i.e., the delivery of messages among processes may be delayed arbitrarily and the processes have no synchronized clocks. Every process is identified by a name, but such identifiers are not made explicit. A protocol for $\mathcal {P}$ consists of a collection of programs with instructions for all processes. Protocols are presented in a modular way using the event-based notation of Cachin et al. [28].

*Executions and faults* An *execution* starts with all processes in a special initial state; subsequently the processes repeatedly trigger events, react to events, and change their state through computation steps. Every execution is *fair* in the sense that, informally, processes do not halt prematurely when there are still steps to be taken or events to be delivered (we refer to the standard literature for a formal definition [29]).

A process that follows its protocol during an execution is called *correct*. On the other hand, a *faulty* process may crash or even deviate arbitrarily from its specification, e.g., when *corrupted* by an adversary; such processes are also called *Byzantine*. We consider only Byzantine faults here and assume for simplicity that the faulty processes fail right at the start of an execution.

*Functionalities* A *functionality* is an abstraction of a distributed computation, either a primitive that may be used by the processes or a service that they will provide. Every functionality in the system is specified through its *interface*, containing the events that it exposes to protocol implementations that may call it, and its *properties*, which define its behavior. A process may react to a received event by changing their state and triggering further events.

There are two kinds of events in an interface: *input events* that the functionality receives from other abstractions, typically to invoke its services, and *output events*, through which the functionality delivers information or signals a condition to a process. The behavior of a functionality is usually stated through a number of properties or through a sequential implementation.

Multiple functionalities may be composed together modularly. In a modular protocol implementation, in particular, every process executes the program instructions of the protocol implementations for all functionalities in which it participates.

*Links* We assume there is a low-level functionality for sending messages over point-to-point links between each pair of processes. In a protocol, this functionality is accessed through the events of “sending a message” and “receiving a message.” Point-to-point messages are authenticated and delivered reliably among correct processes.

Moreover, we assume FIFO ordering on the reliable point-to-point links for every pair of correct processes. This means that if a correct process has “sent” a message $m_1$ and subsequently “sent” a message $m_2$, then every correct process does not “receive” $m_2$ unless it has earlier also “received” $m_1$. FIFO-ordered links are actually a very common assumption. Protocols that guarantee FIFO order on top of (unordered) reliable point-to-point links are well-known and simple to implement [28, 30]. We remark that there is only one FIFO-ordered reliable point-to-point link functionality in the model; hence, FIFO order holds among the messages exchanged by the implementations for *all* functionalities used by a protocol.

*Idealized digital signatures* A *digital signature scheme* provides two operations, $\textit{sign}_i$ and $\textit{verify}_i$. The invocation of $\textit{sign}_i$ specifies a process $p_i$ and takes a bit string $m \in \{0,1\}^*$ as input and returns a signature $\sigma \in \{0,1\}^*$ with the response. Only $p_i$ may invoke $\textit{sign}_i$. The operation $\textit{verify}_i$ takes a putative signature $\sigma$ and a bit string *m* as parameters and returns a Boolean value with the response. Its implementation satisfies that $\textit{verify}_i(\sigma ,m)$ returns true for any $i \in [1,n]$ and $m \in \{0,1\}^*$ if and only if $p_i$ has executed $\textit{sign}_i(m)$ and obtained $\sigma$ before; otherwise, $\textit{verify}_i(\sigma ,m)$ returns false. Every process may invoke *verify*.

## Asymmetric Byzantine quorum systems

This section defines asymmetric Byzantine quorum systems and the notions of a guild and a tolerated system, which are used in protocols later. To set the stage, symmetric Byzantine quorum systems are reviewed first.

### Review of symmetric trust

Quorum systems are well-known in settings with symmetric trust. As demonstrated by many applications to distributed systems, ordinary quorum systems [31] and Byzantine quorum systems [1] play a crucial role in formulating resilient protocols that tolerate faults through replication [32]. A quorum system typically ensures a consistency property among the processes in an execution, despite the presence of some faulty processes.

For the model with Byzantine faults, *Byzantine quorum systems* have been introduced by Malkhi and Reiter [1]. This notion is defined with respect to a *fail-prone system* $\mathcal {F} \subseteq 2^{\mathcal {P}}$, a collection of subsets of $\mathcal {P}$, none of which is contained in another, such that some $F \in \mathcal {F}$ with $F \subseteq \mathcal {P}$ is called a *fail-prone set* and contains all processes that may at most fail together in some execution [1]. A fail-prone system is the same as the *basis* of an *adversary structure*, which was introduced independently by Hirt and Maurer [2].

A fail-prone system captures an assumption on the possible failure patterns that may occur. It specifies all maximal sets of faulty processes that a protocol should tolerate in an execution; this means that a protocol designed for $\mathcal {F}$ achieves its properties as long as the set *F* of actually faulty processes satisfies $F \in \mathcal {F} ^*$. Here and from now on, the notation $\mathcal {A} ^*$ for a system $\mathcal {A} \subseteq 2^\mathcal {P}$, denotes the collection of all subsets of the sets in $\mathcal {A}$, that is, $\mathcal {A} ^* = \{ A' | A' \subseteq A, A \in \mathcal {A} \}$.

#### Definition 1

(*Byzantine quorum system* [1]). A *Byzantine quorum system* for $\mathcal {F}$ is a collection of sets of processes $\mathcal {Q} \subseteq 2^{\mathcal {P}}$ where no set is contained in another and each $Q \in \mathcal {Q}$ is called a *quorum*, such the following properties hold:

- *Consistency:* The intersection of any two quorums contains at least one process that is not faulty, i.e.,
  $$\begin{aligned} \forall Q_1, Q_2 \in \mathcal {Q}, \forall F \in \mathcal {F}: \, Q_1 \cap Q_2 \not \subseteq F. \end{aligned}$$
- *Availability:* For any set of processes that may fail together, there exists a disjoint quorum in $\mathcal {Q}$, i.e.,
  $$\begin{aligned} \forall F \in \mathcal {F}: \, \exists Q \in \mathcal {Q}: \, F \cap Q = \emptyset . \end{aligned}$$

The above notion is also known as a *Byzantine dissemination quorum system* [1] and allows a protocol to be designed despite arbitrary behavior of the potentially faulty processes. The notion generalizes the usual threshold failure assumption for Byzantine faults [33], which considers that any set of *f* processes may fail.

We say that a set system $\mathcal {T}$ *dominates* another set system $\mathcal {S}$ if for each $S \in \mathcal {S}$ there is some $T \in \mathcal {T}$ such that $S \subseteq T$ [34]. In this sense, a quorum system for $\mathcal {F}$ is *minimal* whenever it does not dominate any other quorum system for $\mathcal {F}$. A *maximal* set system is defined analogously.

Similarly to the threshold case, where $n > 3f$ processes are needed to tolerate *f* faulty ones in many Byzantine protocols, Byzantine quorum systems can only exist if not “too many” processes fail.

#### Definition 2

($Q^3$-*condition* [1, 2]). A fail-prone system $\mathcal {F}$ satisfies the $Q^3$*-condition*, abbreviated as $Q^3(\mathcal {F})$, whenever it holds

$$\begin{aligned} \forall F_1, F_2, F_3 \in \mathcal {F}: \, \mathcal {P} \not \subseteq F_1 \cup F_2 \cup F_3. \end{aligned}$$

In other words, $Q^3(\mathcal {F})$ means that no *three* fail-prone sets together cover the whole system of processes. A $Q^k$-condition can be defined like this for any $k \ge 2$ [2].

The following result of Malkhi and Reiter [1, Theorem 5.4] considers the *bijective complement* of a process set $\mathcal {S} \subseteq 2^{\mathcal {P}}$, which is defined as $\overline{\mathcal {S}} = \{ \mathcal {P} {\setminus } S | S \in \mathcal {S} \}$, and turns $\mathcal {F}$ into a Byzantine quorum system. A related theorem was formulated also by Hirt and Maurer [2].

#### Lemma 1

Given a fail-prone system $\mathcal {F}$, a Byzantine quorum system for $\mathcal {F}$ exists if and only if $Q^3(\mathcal {F})$.

In particular, if $Q^3(\mathcal {F})$ holds, then $\overline{\mathcal {F}}$, the bijective complement of $\mathcal {F}$, is a Byzantine quorum system.

The quorum system $\mathcal {Q} = \overline{\mathcal {F}}$ is called the *canonical quorum system* of $\mathcal {F}$. According to the duality between $\mathcal {Q}$ and $\mathcal {F}$, properties of $\mathcal {F}$ are sometimes ascribed to $\mathcal {Q}$ as well. However, note that the canonical quorum system is not always minimal. For instance, if $\mathcal {F}$ consists of all sets of $f \ll n/3$ processes, then each quorum in the canonical quorum system has $n-f$ members, but also the family of all subsets of $\mathcal {P}$ with $\lceil \frac{n+f+1}{2} \rceil < n-f$ processes forms a quorum system.

*Core sets* A *core set* *C* for $\mathcal {F}$ is a minimal set of processes that contains at least one correct process in every execution. More precisely, $C \subseteq \mathcal {P}$ is a core set whenever (1) for all $F \in \mathcal {F}$, it holds $\mathcal {P} {\setminus } F \cap C \ne \emptyset$ (and, equivalently, $C \not \subseteq F$) and (2) for all $C' \subsetneq C$, there exists $F \in \mathcal {F}$ such that $\mathcal {P} {\setminus } F \cap C' = \emptyset$ (and, equivalently, $C' \subseteq F$). With the threshold failure assumption, every set of $f+1$ processes is a core set. A *core-set system* $\mathcal {C}$ is the minimal collection of all core sets, in the sense that no set in $\mathcal {C}$ is contained in another.

Core sets can be complemented by *survivor sets*, as shown by Junqueira et al. [35]. This yields a dual characterization of resilient distributed protocols, which parallels ours using fail-prone sets and quorums.

*Kernels* Given a symmetric Byzantine quorum system $\mathcal {Q}$, we define a *kernel* *K* as a minimal set of processes that overlaps with every quorum. A kernel generalizes the notion of a *core set* [36].

#### Definition 3

(*Kernel system*). A set $K \subseteq \mathcal {P}$ is a *kernel* of a quorum system $\mathcal {Q}$ if an only if

$$\begin{aligned} \forall Q \in \mathcal {Q}: \, K \cap Q \ne \emptyset \end{aligned}$$

and

$$\begin{aligned} \forall K' \subsetneq K: \, \exists ~Q \in \mathcal {Q}: \, Q \cap K' = \emptyset . \end{aligned}$$

We also define the *kernel system* $\mathcal {K}$ of $\mathcal {Q}$ to be the set of all kernels of $\mathcal {Q}$.

For example, under a threshold failure assumption where any *f* processes may fail, every set of $\big \lfloor \frac{n-f+1}{2}\big \rfloor$ processes is a kernel. In particular, $n=3f+1$ if and only if every kernel has $f+1$ processes.

The definition of a kernel is related to that of a core set in the following sense.

#### Lemma 2

Let $\mathcal {F}$ be a fail-prone system and $\mathcal {Q} = \overline{\mathcal {F}}$ be the canonical quorum system of $\mathcal {F}$. Then the kernel system of $\mathcal {Q}$ is the same as the core-set system for $\mathcal {F}$.

#### Proof

Consider a kernel system $\mathcal {K}$ of a Byzantine quorum system $\mathcal {Q}$. By definition, the following two properties hold with respect to every kernel $K \in \mathcal {K}$:

(i) For every quorum *Q* in $\mathcal {Q}$, the intersection with the kernel *K* is non-empty, i.e., $K \cap Q \ne \emptyset$.
(ii) For any proper subset $K'$ of *K*, there exists a quorum *Q* in $\mathcal {Q}$ such that $K'$ does not intersect with *Q*, i.e., $Q \cap K' = \emptyset$.

Given the canonical quorum system $\mathcal {Q}$ derived from the fail-prone system $\mathcal {F}$, by definition of canonical quorum system of $\mathcal {F}$ we have that for every *Q* in $\mathcal {Q}$, there exists a unique fail-prone set *F* in $\mathcal {F}$ such that *Q* is precisely the complement of *F* within $\mathcal {P}$, that is, $Q = \mathcal {P} \setminus F$. Consequently, the concepts of a kernel and a core set are equivalent in this context, as a core set is defined with respect to sets of the form $\mathcal {P} {\setminus } F$. $\square$

#### Lemma 3

Let $\mathcal {F}$, $\mathcal {Q}$, and $\mathcal {K}$ be a fail-prone system, a Byzantine quorum system for $\mathcal {F}$, and the kernel system of $\mathcal {Q}$, respectively. Then, for every quorum $Q \in \mathcal {Q}$, there exists a kernel $K \in \mathcal {K}$ such that $K \subseteq Q$.

#### Proof

Consider the quorum system $\mathcal {Q}$ for $\mathcal {F}$. Let *F* be any such fail-prone set in $\mathcal {F}$. For a given quorum $Q \in \mathcal {Q}$, define the set $K = Q \setminus F$. By definition, *K* is a subset of *Q*, i.e., $K \subseteq Q$. The consistency property of the Byzantine quorum system now implies that any two quorums $Q, Q' \in \mathcal {Q}$ have an intersection $Q \cap Q'$ that is not fully contained within *F*. Therefore, *K* intersects with $Q'$ since $(Q {\setminus } F) \cap Q' = K \cap Q'$ is not empty. This property holds for every $Q' \in \mathcal {Q}$ and confirms that *K* intersects with every quorum in $\mathcal {Q}$. As such, *K* satisfies the first property of a kernel of $\mathcal {Q}$.

For the second property, minimality, let us consider such a *K*. To construct a kernel contained in *Q*, we progressively remove elements from *K*, ensuring that the resultant subset retains the property of intersection with all quorums. This process terminates with a subset $K^*$, which cannot be reduced further without losing the intersection property. The minimality of $K^*$ is guaranteed by the contradiction that arises from the assumption that a proper subset of $K^*$ could intersect with all quorums, as this would violate the termination of our removal process. Therefore, $K^*$ is a kernel by definition since it is the minimal intersecting set with every quorum in $\mathcal {Q}$, and it is contained within the original quorum *Q* from which we subtracted *F*. This shows that $K^*$ is a kernel of *Q*. $\square$

### Asymmetric trust

In our model with asymmetric trust, every process is free to make its own trust assumption and to express this with a fail-prone system. Hence, an *asymmetric fail-prone system* $\mathbb {F} = [\mathcal {F} _1, \dots , \mathcal {F} _n]$ consists of an array of fail-prone systems, where $\mathcal {F} _i$ denotes the trust assumption of $p_i$. One often assumes $p_i \not \in F_i$ for practical reasons, but this is not necessary. This notion has earlier been formalized by Damgård et al. [3].

#### Definition 4

(*Asymmetric Byzantine quorum system*). An *asymmetric Byzantine quorum system* for $\mathbb {F}$ is an array of collections of sets $\mathbb {Q} = [\mathcal {Q} _1, \dots , \mathcal {Q} _n]$, where $\mathcal {Q} _i \subseteq 2^{\mathcal {P}}$ for $i \in [1,n]$. The set $\mathcal {Q} _i \subseteq 2^{\mathcal {P}}$ is called the *quorum system of* $p_i$ and any set $Q_i \in \mathcal {Q} _i$ is called a *quorum (set) for* $p_i$. It satisfies:

- *Consistency:* The intersection of two quorums for any two processes contains at least one process for which either process assumes that it is not faulty, i.e.,
  $$\begin{aligned} \forall i,j \in [1,n],&\forall Q_i \in \mathcal {Q} _i, \forall Q_j \in \mathcal {Q} _j,\\&\forall F_{ij} \in {\mathcal {F} _i}^* \cap {\mathcal {F} _j}^*: \, Q_i \cap Q_j \not \subseteq F_{ij}. \end{aligned}$$
- *Availability:* For any process $p_i$ and any set of processes that may fail together according to $p_i$, there exists a disjoint quorum for $p_i$ in $\mathcal {Q} _i$, i.e.,
  $$\begin{aligned} \forall i \in [1,n], \forall F_i \in \mathcal {F} _i: \, \exists Q_i \in \mathcal {Q} _i: \, F_i \cap Q_i = \emptyset . \end{aligned}$$

Recall that the consistency condition for a (symmetric) Byzantine quorum system requires that at least one process in the intersection of every two quorums is correct. In the asymmetric case, quorums are subjective and defined according to the quorum system for each process. The asymmetric consistency property states that in the intersection of every two subjective quorums of two processes there exists at least one process that is correct according to one of the two processes. On the other hand, the availability condition in the above definition is a direct extension of the symmetric case, since it considers the quorum system of each process separately. We remark that availability suffices for implementing some protocols but a stronger assumption (i.e., the existence of a guild, introduced below) is needed for others.

The existence of asymmetric quorum systems can be characterized with a property that generalizes the $Q^3$-condition for the underlying asymmetric fail-prone systems as follows.

#### Definition 5

($B^3$-*condition*). An asymmetric fail-prone system $\mathbb {F}$ satisfies the $B^3$*-condition*, abbreviated as $B^3(\mathbb {F})$, whenever it holds that

$$\begin{aligned} \forall i,j \in [1,n],&\forall F_i \in \mathcal {F} _i, \forall F_j\in \mathcal {F} _j,\\&\forall F_{ij} \in {\mathcal {F} _i}^*\cap {\mathcal {F} _j}^*: \, \mathcal {P} \not \subseteq F_i \cup F_j \cup F_{ij} \end{aligned}$$

The following result is the generalization of Lemma 1 for asymmetric quorum systems; it was stated by Damgård et al. [3] without proof.

#### Theorem 4

An asymmetric fail-prone system $\mathbb {F}$ satisfies $B^3(\mathbb {F})$ if and only if there exists an asymmetric quorum system for $\mathbb {F}$.

#### Proof

Suppose that $B^3(\mathbb {F})$. We let $\mathbb {Q} = [\mathcal {Q} _1, \dots , \mathcal {Q} _n]$, where $\mathcal {Q} _i = \overline{\mathcal {F} _i}$ is the canonical quorum system of $\mathcal {F} _i$, and show that $\mathbb {Q}$ is an asymmetric quorum system. Indeed, let $Q_i \in \mathcal {Q} _i$, $Q_j \in \mathcal {Q} _j$, and $F_{ij} \in {\mathcal {F} _i}^* \cap {\mathcal {F} _j}^*$ for any *i* and *j*. Then $F_i = \mathcal {P} {\setminus } Q_i \in \mathcal {F} _i$ and $F_j = \mathcal {P} {\setminus } Q_j \in \mathcal {F} _j$ by construction, and therefore, $F_i \cup F_j \cup F_{ij} \not = \mathcal {P}$ holds according to $B^3(\mathbb {F})$. This means there is some $p_k \in \mathcal {P} {\setminus } (F_i \cup F_j \cup F_{ij})$. Because $p_k \not \in F_i$, it holds $p_k \in Q_i$ and analogously $p_k \in Q_j$. This implies in turn that $p_k \in Q_i \cap Q_j$ but $p_k \notin F_{ij}$ and proves the consistency condition. The availability property holds by construction of the canonical quorum systems.

To show the reverse direction, let $\mathbb {Q}$ be a candidate asymmetric Byzantine quorum system for $\mathbb {F}$ that satisfies availability and assume towards a contradiction that $B^3(\mathbb {F})$ does not hold. We show that consistency cannot be fulfilled for $\mathbb {Q}$. By our assumption there are sets $F_i, F_j, F_{ij}$ in $\mathbb {F}$ such that $F_i \cup F_j \cup F_{ij} = \mathcal {P}$, which means also that $\mathcal {P} {\setminus } (F_i \cup F_j) \subseteq F_{ij}$. The availability condition for $\mathbb {Q}$ then implies that there are sets $Q_i \in \mathcal {Q} _i$ and $Q_j \in \mathcal {Q} _j$ with $F_i \cap Q_i = \emptyset$ and $F_j \cap Q_j = \emptyset$. Now for every $p_k \in Q_i \cap Q_j$ it holds that $p_k \notin F_i \cup F_j$ by availability and therefore $p_k \in \mathcal {P} {\setminus } (F_i \cup F_j)$. Taken together this means that $Q_i \cap Q_j \subseteq \mathcal {P} {\setminus } (F_i \cup F_j) \subseteq F_{ij}$. Hence, $\mathbb {Q}$ does not satisfy the consistency condition and the statement follows. $\square$

*Asymmetric core sets and kernels* Let $\mathbb {F} = [ \mathcal {F} _1, \dots , \mathcal {F} _n ]$ be an asymmetric fail-prone system. An *asymmetric core-set system* $\mathbb {C}$ is an array of collections of sets $[ \mathcal {C} _1, \dots , \mathcal {C} _n ]$ such that each $\mathcal {C} _i$ is a core set system for the fail-prone system $\mathcal {F} _i$. We call a set $C_i \in \mathcal {C} _i$ a *core set for* $p_i$.

**Fig. 1:**
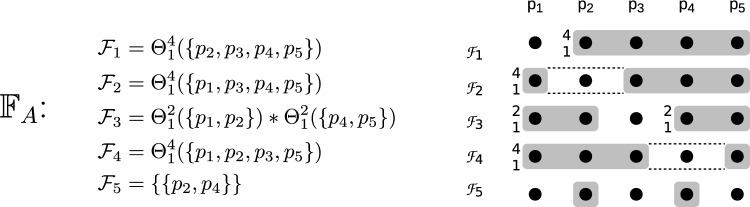
The asymmetric fail-prone system $\mathbb {F} _A$ with five processes described in Example 1. The notation $_{k}^{n}$ in front of a fail-prone set stands for *k* out of the *n* processes in the set, and the operator $*$ for two sets satisfies $\mathcal {A} *\mathcal {B} = \{ A \cup B | A \in \mathcal {A}, B \in \mathcal {B} \}$

Given an asymmetric quorum system $\mathbb {Q}$ for $\mathbb {F}$, an *asymmetric kernel system* for $\mathbb {Q}$ is defined analogously as the array $\mathbb {K} = [\mathcal {K} _1, \dots , \mathcal {K} _n]$ that consists of the kernel systems for all processes in $\mathcal {P}$. A set $K_i \in \mathcal {K} _i$ is called a *kernel for* $p_i$. This means that every kernel for $p_i$ has a non-empty intersection with every quorum of $p_i$.

*Naïve and wise processes* Recall that the guarantees of quorum-based protocols apply to *correct* processes only, but not to faulty ones. The faults or corruptions occurring in a protocol execution with an underlying quorum system induce a set *F* of actually *faulty processes*. However, no process knows *F* and this information is only available to an observer outside the system. With a traditional quorum system $\mathcal {Q}$ designed for a fail-prone set $\mathcal {F}$, the guarantees of a protocol usually hold as long as $F \in \mathcal {F} ^*$, and if *F* is not contained in $\mathcal {F} ^*$, no useful properties can be derived for any process.

With asymmetric quorums, we further distinguish between two kinds of correct processes, depending on whether they considered *F* in their trust assumption or not. Given a protocol execution, the processes are therefore partitioned into three types:

- *Faulty:* A process $p_i \in F$ is *faulty*.
- *Naïve:* A correct process $p_i$ for which $F \not \in {\mathcal {F} _i}^*$ is called *naïve*.
- *Wise:* A correct process $p_i$ for which $F \in {\mathcal {F} _i}^*$ is called *wise*.

The naïve processes are new for the asymmetric case, as all correct processes are wise under a symmetric trust assumption. Protocols for asymmetric quorums cannot guarantee the same properties for naïve processes as for wise ones, since the naïve processes may have the “wrong friends.” In one formalization of the Stellar protocol, correct nodes that find themselves in a similar situation have been called “befouled” [13].

#### Example 1

We define an example of asymmetric fail-prone system $\mathbb {F} _A$ on $\mathcal {P} = \{p_1, p_2, p_3, p_4, p_5\}$. The notation $\Theta ^n_k(\mathcal {S})$ for a set $\mathcal {S}$ with *n* elements denotes the “threshold” combination operator and enumerates all subsets of $\mathcal {S}$ of cardinality *k*. W.l.o.g. every process trusts itself. The diagram in Fig. 1 shows fail-prone sets as shaded areas and the notation $_{k}^{n}$ in front of a fail-prone set stands for *k* out of the *n* processes in the set.

The operator $*$ for two sets satisfies $\mathcal {A} *\mathcal {B} = \{ A \cup B | A \in \mathcal {A}, B \in \mathcal {B} \}$.

As one can verify in a straightforward way, $B^3(\mathbb {F} _A)$ holds. Let $\mathbb {Q} _A$ be the canonical asymmetric quorum system for $\mathbb {F} _A$. Note that since $\mathbb {F} _A$ contains the fail-prone systems of $p_3$ and $p_5$ that permit two faulty processes each, this fail-prone system cannot be obtained as a special case of $\Theta ^5_1(\{p_1, p_2, p_3, p_4, p_5\})$. When $F = \{p_2, p_4\}$, for example, then processes $p_3$ and $p_5$ are wise and $p_1$ is naïve.

*Guilds* If too many processes are naïve or even fail during a protocol run with asymmetric quorums, then protocol properties cannot be ensured. A *guild* is a set of wise processes that contains at least one quorum for each member; by definition this quorum consists only of wise processes. A guild ensures liveness and consistency for typical protocols. This generalizes from protocols with symmetric trust, where the correct processes in every execution form a quorum by definition. A guild represents a group of influential and well-connected wise processes, like in the real world.

#### Definition 6

(*Guild*). Given a fail-prone system $\mathbb {F}$, an asymmetric quorum system $\mathbb {Q}$ for $\mathbb {F}$, and a protocol execution with faulty processes *F*, a *guild* $\mathcal {G}$ *for* *F* *and* $\mathbb {Q}$ satisfies two properties:

*Wisdom:* $\mathcal {G}$ is a set of wise processes:
  $$\begin{aligned} \forall p_i \in \mathcal {G}:\, F \in {\mathcal {F} _i}^*. \end{aligned}$$
*Closure:* $\mathcal {G}$ contains a quorum for each of its members:
  $$\begin{aligned} \forall p_i \in \mathcal {G}:\, \exists Q_i \in \mathcal {Q} _i:\, Q_i \subseteq \mathcal {G}. \end{aligned}$$

A guild is related to an “intact set” in the Stellar consensus protocol [13, 19], but the two notions differ in how they are defined. Observe that the union of two guilds is again a guild, since the union consists only of wise processes and contains again a quorum for each member. All guilds overlap, as the next result shows.

#### Lemma 5

In any execution with a guild $\mathcal {G}$, every two guilds intersect.

#### Proof

Let $\mathcal {P}$ be a set of processes, $\mathcal {G}$ be a guild, and *F* be the set of actually faulty processes. Furthermore, suppose that there is another guild $\mathcal {G} '$. Let $p_i \in \mathcal {G}$ and $p_j \in \mathcal {G} '$ be two processes and consider a quorum $Q_i \subseteq \mathcal {G}$ for $p_i$ and a quorum $Q_j \subseteq \mathcal {G} '$ for $p_j$. From the definition of an asymmetric quorum system it must hold $Q_i \cap Q_j \nsubseteq F$, with $Q_i \cap Q_j \ne \emptyset$ and $F \in {\mathcal {F} _i}^* \cap {\mathcal {F} _j}^*$. It follows that there exists a wise process $p_k \in Q_i \cap Q_j$ with $p_k \in \mathcal {G}$ and $p_k \in \mathcal {G} '$. Notice also that $\mathcal {G}$ and $\mathcal {G} '$ both contain a quorum for $p_k$. $\square$

It follows that every execution with a guild contains a unique *maximal guild* $\mathcal {G} _{\max }$. The next lemma shows that if a guild exists, no quorum for any process contains only faulty processes.

#### Lemma 6

Let $\mathcal {G} _{\text {max}}$ be the maximal guild for a given execution and let $\mathbb {Q}$ be the canonical asymmetric quorum system. Then, there cannot be a quorum $Q_j \in \mathcal {Q}_j$ for any process $p_j$ consisting only of faulty processes.

#### Proof

Given an execution with *F* as set of faulty processes, suppose there is a guild $\mathcal {G} _{\text {max}}$. This means that for every process $p_i \in \mathcal {G} _{\text {max}}$, a quorum $Q_i \subseteq \mathcal {G} _{\text {max}}$ exists such that $Q_i \cap F = \emptyset$. It follows that for every $p_i \in \mathcal {G} _{\text {max}}$, there is a set $F_i \in \mathcal {F}_i$ such that $F \subseteq F_i$. Recall that since $\mathbb {Q}$ is a quorum system, $B^3(\mathbb {F})$ holds. From Definition 5, we have that for all $i,j \in [1,n]$, all $F_i \in \mathcal {F} _i, \forall F_j\in \mathcal {F} _j$, and all $F_{ij} \in {\mathcal {F} _i}^*\cap {\mathcal {F} _j}^*$, it holds $\mathcal {P} \not \subseteq F_i \cup F_j \cup F_{ij}$.

Towards a contradiction, assume that there is a process $p_j$ such that there exists a quorum $Q_j \in \mathcal {Q}_j$ for $p_j$ with $Q_j = F$. This implies that there exists $F_j \in \mathcal {F}_j$ such that $F_j = \mathcal {P} \setminus F$.

Let $F_i$ be the fail-prone system of $p_i \in \mathcal {G} _{\text {max}}$ such that $F \subseteq F_i$ and let $F_j = \mathcal {P} \setminus F$ as just defined. Then, $F_i \cup F_j \cup F_{ij} = \mathcal {P}$. This follows from the fact that $F_i$ contains *F* and that $F_j = \mathcal {P} {\setminus } F$. This contradicts the $B^3$-condition for $\mathbb {F}$. $\square$

#### Lemma 7

Let $\mathcal {G} _{\text {max}}$ be the maximal guild for a given execution and let $p_i$ be any correct process. Then, every quorum for $p_i$ contains at least one process in $\mathcal {G} _{\text {max}}$.

#### Proof

The claim naturally derives from the consistency property of an asymmetric quorum system. Consider any correct process $p_i$ and one of its quorums, $Q_i \in \mathcal {Q} _i$. For any process $p_j \in \mathcal {G} _{\text {max}}$, let $Q_j$ be a quorum of $p_j$ such that $Q_j \subseteq \mathcal {G} _{\text {max}}$, which exists because $\mathcal {G} _{\text {max}}$ is a guild. Then, the quorum consistency property implies that $Q_i \cap Q_j \ne \emptyset$. Thus, $Q_i$ contains a process in the maximal guild. $\square$

Finally, we show with an example that it is possible for a wise process to be outside the maximal guild.

#### Example 2

**Fig. 2:**
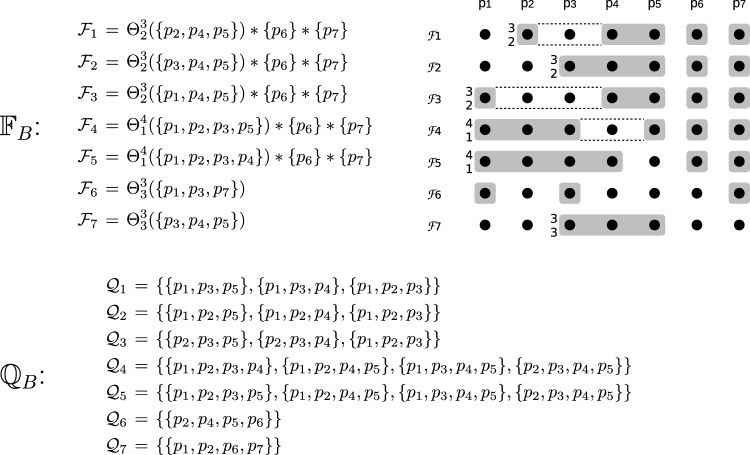
A seven-process asymmetric fail-prone system $\mathbb {F} _B$, shown above, and the corresponding canonical asymmetric quorum system $\mathbb {Q} _B$, shown below. See Example 2 for a discussion

Figure 2 shows a seven-process asymmetric quorum system $\mathbb {Q} _B$, defined through its fail-prone system $\mathbb {F} _B$. One can verify that $B^3(\mathbb {F} _B)$ holds and that $\mathbb {Q} _B$ is the canonical quorum system for $\mathbb {F} _B$.

With $F = \{p_4, p_5\}$, for instance, processes $p_1, p_2, p_3$ and $p_7$ are wise, $p_6$ is naïve, and $\mathcal {G} _{\text {max}} = \{p_1, p_2, p_3\}$. It follows that process $p_7$ is wise but outside the guild $\mathcal {G} _{\text {max}}$, because the unique maximal quorum in $\mathcal {Q} _7$ contains the naïve process $p_6$.

Lemma 7 reveals the interesting result that for an execution with a guild, each quorum of every correct process $p_i$ contains at least one process that is also in the maximal guild $\mathcal {G} _{\text {max}}$. Since a kernel for $p_i$ is a process set that has some member in common with every quorum of $p_i$, this implies that $\mathcal {G} _{\text {max}}$ contains a kernel for $p_i$.

#### Corollary 8

In every execution with a guild, the maximal guild $\mathcal {G} _{\text {max}}$ contains a kernel for every correct process.

It follows that whenever all processes in the maximal guild send some particular message, then every correct process will eventually receive this message from all processes in one of its kernels. This is exploited by protocols that use kernels, such as Algorithm 4 (in Sect. 6).

A guild can also be seen as a set of sufficiently many wise processes that allow a protocol to make progress, in the following sense.

#### Lemma 9

Consider an execution, in which the processes in *F* are faulty and let $\mathcal {G}_{\text {max}}$ be the maximal guild for *F*. Let *A* be a superset of *F* that is disjoint from $\mathcal {G}_{\text {max}}$, i.e., $F \subseteq A \subseteq \mathcal {P} {\setminus } \mathcal {G}_{\text {max}}$.

Then, in any execution where the processes in *A* fail, $\mathcal {G}_{\text {max}}$ is also the maximal guild for *A*.

#### Proof

Let $\mathcal {G}_{\text {max}}$ be the maximal guild in an execution with set of faulty processes $F \subseteq \mathcal {P}{\setminus } \mathcal {G}_{\text {max}}$. By definition of a guild, $\mathcal {G}_{\text {max}}$ contains a quorum for each of its members. This means that there exists a quorum $Q_i$ for every $p_i \in \mathcal {G}_{\text {max}}$ such that $Q_i \cap ~F = \emptyset .$ This also implies that for every set $A \supseteq F$, with $A \subseteq \mathcal {P} {\setminus } \mathcal {G}_{\text {max}}$, we have that $Q_i \cap ~A = \emptyset$, and the lemma follows. $\square$

Given the importance of a guild for an asymmetric Byzantine quorum system, we introduce the following notion.

#### Definition 7

(*Tolerated system*). Given an asymmetric Byzantine quorum system $\mathbb {Q}$ and an execution with faulty processes *F*, a set of processes *T* is called *tolerated (by* $\mathbb {Q}$ *)* if a non-empty guild $\mathcal {G}$ for *F* and $\mathbb {Q}$ exists such that $T = \mathcal {P} \setminus \mathcal {G}$.

The *tolerated system* $\mathcal {T}$ of an asymmetric Byzantine quorum system $\mathbb {Q}$ is the maximal collection of tolerated sets, where *F* ranges over all possible executions.

Intuitively, the tolerated system of an asymmetric Byzantine quorum system reflects its resilience: even when all processes in a tolerated set fail, there still exists a non-empty guild. Therefore, the tolerated system characterizes the executions in which some processes will be able to operate correctly and make progress (where progress is defined by the protocol they are running). In that sense, the tolerated system of an asymmetric Byzantine quorum system can be seen as a counterpart of the fail-prone system in the symmetric model.

Notice that the tolerated system is a global notion emerging from the subjective trust choices of the participating processes; any process that knows the fail-prone and quorum systems of all processes can calculate it. We remark that the tolerated system is a central concept for composing asymmetric Byzantine quorum system, as shown by Alpos et al. [37].

The following lemma shows that the tolerated system $\mathcal {T}$ of a canonical asymmetric Byzantine quorum system is itself a symmetric fail-prone system. In particular, $\tau$ builds a connection to symmetric quorum-based protocols. This property will be used in Sect. 7 to construct an asymmetric common coin protocol.

#### Lemma 10

Let $\mathbb {Q}$ be an asymmetric Byzantine quorum system among processes $\mathcal {P}$ with asymmetric fail-prone system $\mathbb {F} = \overline{\mathbb {Q}}$, i.e., such that $\mathbb {Q}$ is a canonical asymmetric Byzantine quorum system, and let $\mathcal {T}$ be the tolerated system of $\mathbb {Q}$. If $B^3(\mathbb {F})$, then $Q^3(\mathcal {T})$.

#### Proof

Towards a contradiction, let us assume that $\mathcal {T}$ does not satisfy the $Q^3$-condition. This means that there exist $T_1, T_2, T_3 \in \mathcal {T}$ such that $T_1 \cup T_2 \cup T_3 = \mathcal {P}$. Also, let $\mathcal {G} _1, \mathcal {G} _2, \mathcal {G} _3$ be the corresponding guilds, i.e., $\mathcal {G} _1 = \mathcal {P} {\setminus } T_1, \mathcal {G} _2 = \mathcal {P} {\setminus } T_2$ and $\mathcal {G} _3 = \mathcal {P} {\setminus } T_3$. By assumption, every guild contains at least one process and at least one quorum for this process is fully contained in the guild. By the consistency property of an asymmetric Byzantine quorum system, these quorums must intersect pairwise, hence the guilds also intersect pairwise. This means that there exist processes $p_{i} \in \mathcal {G} _1 \cap \mathcal {G} _2$ and $p_{j} \in \mathcal {G} _2 \cap \mathcal {G} _3$. Now, because $p_i$ is a member of $\mathcal {G} _1$, we can make the following reasoning: $p_i$ has a quorum $Q_i \in \mathcal {Q} _i$ such that $Q_i \subseteq \mathcal {G} _1$, the quorum system is canonical, so $p_i$ has a fail-prone set $F_i = \mathcal {P} {\setminus } Q_i \in \mathcal {F} _i$, thus we get $T_1 \subseteq F_i$, i.e., $T_1 \in \mathcal {F} _i$. With similar reasoning, we get $T_2 \in \mathcal {F} _i$ (because $p_i \in \mathcal {G} _2$), $T_2 \in \mathcal {F} _j$ (because $p_j \in \mathcal {G} _2$), and $T_3 \in \mathcal {F} _j$ (because $p_j \in \mathcal {G} _3$). But this is a contradiction because $p_i$ and $p_j$ with fail-prone sets $T_1, T_2$, and $T_3$ violate the $B^3$-condition in $\mathbb {Q}$.

$$\square $$

## Shared memory

This section illustrates a first application of asymmetric quorum systems: how to emulate shared memory, represented by a *register*. Maintaining a shared register reliably in a distributed system subject to faults is perhaps the most fundamental task for which ordinary, symmetric quorum systems have been introduced, in the models with crashes [38] and with Byzantine faults [1].

### Definitions

*Operations and precedence* For the particular *shared-object* functionalities considered here, the processes interact with an object $\Lambda$ through *operations* provided by $\Lambda$. Operations on objects take time and are represented by two events occurring at a process, an *invocation* and a *response*. The *history* of an execution *h* consists of the sequence of invocations and responses of $\Lambda$ occurring in *h*. An operation is *complete* in a history if it has a matching response.

An operation *o* *precedes* another operation $o'$ in a sequence of events *h*, denoted $o <_h o'$, whenever *o* completes before $o'$ is invoked in *h*. A sequence of events $\pi$ *preserves the real-time order* of a history *h* if for every two operations *o* and $o'$ in $\pi$, if $o <_h o'$ then $o<_\pi o'$. Two operations are *concurrent* if neither one of them precedes the other. A sequence of events is *sequential* if it does not contain concurrent operations. An execution on a shared object is *well-formed* if the events at each process are alternating invocations and matching responses, starting with an invocation.

*Semantics* A *register* with domain $\mathcal {X}$ provides two operations: $\textit{write}(x)$, which is parameterized by a value $x \in \mathcal {X}$ and outputs a token ack when it completes; and $\textit{read}$, which takes no parameter for invocation but outputs a value $x \in \mathcal {X}$ upon completion.

We consider a *single-writer* (or *SW*) register, where only a designated process $p_w$ may invoke *write*, and permit *multiple readers* (or *MR*), that is, every process may execute a *read* operation. The register is initialized with a special value $x_0$, which is written by an imaginary *write* operation that occurs before any process invokes operations. We consider *regular* semantics under concurrent access [39]; the extension to other forms of concurrent memory, including an atomic register, proceeds analogously.

It is customary in the literature to assume $p_w$ writes every value in $\mathcal {X}$ at most once. Furthermore, the writer and the reader are correct; with asymmetric quorums we assume explicitly that readers and writers are *wise*. We illustrate below why one cannot extend the guarantees of the register to naïve processes.

#### Definition 8

(*Asymmetric Byzantine SWMR regular register*) A protocol emulating an *asymmetric SWMR regular register* satisfies:

- *Liveness:* If a wise process *p* invokes an operation on the register, *p* eventually completes the operation.
- *Safety:* Every *read* operation of a wise process that is not concurrent with a *write* returns the value written by the most recent, preceding *write* of a wise process; furthermore, a *read* operation of a wise process concurrent with a *write* of a wise process may also return the value that is written concurrently.

### Protocol with authenticated data

In Algorithm 1, we describe a protocol for emulating a regular SWMR register with an asymmetric Byzantine quorum system, for a designated writer $p_w$ and a reader $p_r \in \mathcal {P}$. The protocol uses *data authentication* implemented with digital signatures. This protocol is the same as the classic one of Malkhi and Reiter [1] that uses a Byzantine dissemination quorum system and where processes send messages to each other over point-to-point links. The difference lies in the individual choices of quorums by the processes and that it ensures safety and liveness for wise processes.

In more detail, every process stores a triple $(\textit{ts}, v, \sigma )$, which consists of a timestamp *ts*, a value *v*, and a signature $\sigma$. The idea is that the writer maintains a timestamp that increases with every *write* operation. The writer $p_w$ signs the timestamp/value pair and sends it in a message together with the signature to the processes, who will store the data if the timestamp within the received message is higher than the timestamp *ts* stored locally. A process then responds to $p_w$ with an ack message. The change from the classic protocol is the writer $p_w$ obtains ack messages from all processes in a quorum $Q_w \in \mathcal {Q} _w$ for itself. The reader $p_r$ sends a read message to all processes. It then waits to receive responses, which carry a triple of value, timestamp, and signature such that the signature is valid, from processes in a quorum $Q_r$ for $p_r$. The returned value is the one from the triple with the highest timestamp.
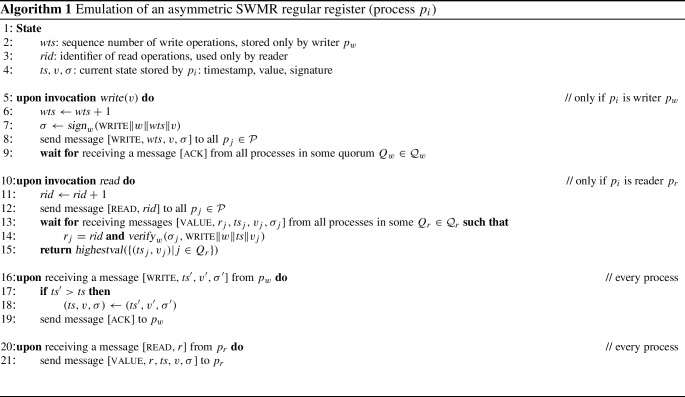


The function $\textit{highestval}(S)$ takes a set of timestamp/value pairs *S* as input and outputs the value in the pair with the largest timestamp, i.e., *v* such that $(\textit{ts}, v) \in S$ and $\forall (\textit{ts} ', v') \in S: \textit{ts} ' < \textit{ts} \vee (\textit{ts} ', v') = (\textit{ts}, v)$. Note that this *v* is unique in Algorithm 1 because $p_w$ is correct. The protocol uses digital signatures, modeled by operations $\textit{sign}_i$ and $\textit{verify}_i$, as introduced earlier.

#### Theorem 11

Algorithm 1 emulates an asymmetric Byzantine SWMR regular register.

#### Proof

First we show liveness for wise writer $p_w$ and reader $p_r$, respectively. Since $p_w$ is wise by assumption, $F \in {\mathcal {F} _w}^*$, and by the availability condition of the quorum system there is $Q_w \in \mathcal {Q} _w$ with $F \cap Q_w = \emptyset$. Therefore, the writer will receive sufficiently many $[\textsc {ack}]$ messages and the *write* will return. As $p_r$ is wise, $F \in {\mathcal {F} _r}^*$, and by the analogous condition, there is $Q_r \in \mathcal {Q} _r$ with $F \cap Q_r = \emptyset$. Because $p_w$ is correct and by the properties of the signature scheme, all responses from processes $p_j \in Q_r$ satisfy the checks and *read* returns.

Regarding safety, it is easy to observe that any value output by *read* has been written in some preceding or concurrent *write* operation, and this even holds for naïve readers and writers. This follows from the properties of the signature scheme; *read* verifies the signature and outputs only values with a valid signature produced by  $p_w$.

We now argue that when both the writer and the reader are wise, then *read* outputs a value of either the last preceding *write* or a concurrent *write* and the protocol satisfies safety for a regular register. On a high level, note that $F \in {\mathcal {F} _w}^* \cap {\mathcal {F} _r}^*$ since both are wise. So if $p_w$ writes to a quorum $Q_w \in \mathcal {Q} _w$ and $p_r$ reads from a quorum $Q_r \in \mathcal {Q} _r$, then by consistency of the quorum system $Q_w \cap Q_r \not \subseteq F$ because $p_w$ and $p_r$ are wise. Hence, there is some correct $p_i \in Q_w \cap Q_r$ that received the most recently written value from $p_w$ and returns it to $p_r$. $\square$

#### Example 3

We show why the guarantees of this protocol with asymmetric quorums hold only for wise readers and writers. Consider $\mathbb {Q} _A$ from the last section and an execution in which $p_2$ and $p_4$ are faulty, and therefore $p_1$ is naïve and $p_3$ and $p_5$ are wise. A quorum for $p_1$ consists of $p_1$ and three processes in $\{ p_2, \dots , p_5\}$; moreover, every process set that contains $p_3$, one of $\{p_1, p_2\}$ and one of $\{p_4, p_5\}$ is a quorum for $p_3$.

We illustrate that if naïve $p_1$ writes, then a wise reader $p_3$ may violate safety. Suppose that all correct processes, especially $p_3$, store timestamp/value/signature triples from an operation that has terminated and that wrote *x*. When $p_1$ invokes $\textit{write}(u)$, it obtains [ack] messages from all processes except $p_3$. This is a quorum for $p_1$. Then $p_3$ runs a *read* operation and receives the outdated values representing *x* from itself ($p_3$ is correct but has not been involved in writing *u*) and also from the faulty $p_2$ and $p_4$. Hence, $p_3$ outputs *x* instead of *u*.

Analogously, with the same setup of every process initially storing a representation of *x* but with wise $p_3$ as writer, suppose $p_3$ executes $\textit{write}(u)$. It obtains [ack] messages from $p_2$, $p_3$, and $p_4$ and terminates. When $p_1$ subsequently invokes *read* and receives values representing *x*, from correct $p_1$ and $p_5$ and from faulty $p_2$ and $p_4$, then $p_1$ outputs *x* instead of *y* and violates safety as a naïve reader.

Since the sample operations are not concurrent, the implication actually holds also for registers with only safe semantics.

### Double-write protocol without data authentication

This section describes a second protocol emulating an asymmetric Byzantine SWMR regular register. In contrast to the previous protocol, it does not use digital signatures for authenticating the data to the reader. Our algorithm generalizes the construction of Abraham et al. [40] and also assumes that only a finite number of write operations occur (*FW-termination*). Furthermore, this algorithm illustrates the use of asymmetric core-set systems in the context of an asymmetric-trust protocol.

This protocol extends Algorithm 1 and every process stores the most recently written timestamp-value pair $(\textit{ts}, v)$. Every *write* operation performs two rounds instead of one, a pre-write round and a write round. In addition to the previous protocol, every process stores the most recently pre-written timestamp-value pair $(\textit{pts}, \textit{pv})$. From the perspective of the writer $p_w$, each round proceeds like the single round in Algorithm 1, except that $p_w$ does not produce a digital signature. In particular, $p_w$ waits in each round for responses that form a quorum $Q_w \in \mathcal {Q} _w$ for itself.

The reader $p_r$ exchanges one round of messages with the processes and waits for responses that form a quorum $Q_r \in \mathcal {Q} _r$ for $p_r$. Every response contains the pre-written and the written timestamp-value pairs from the sending process. The reader collects these in an array *readlist* until the following condition is satisfied. A pair $(\textit{ts} ^*, v^*)$, a core set $C_r$ for $p_r$ of entries in *readlist*, and a quorum $Q_r$ for $p_r$ of entries in *readlist* exist such that (1) the pair $(\textit{ts} ^*, v^*)$ is either the pre-written or the written pair in all entries of *readlist* in $C_r$; and (2) $(\textit{ts} ^*, v^*)$ is the pair with the highest timestamp among the entries in $Q_r$. Intuitively, the initial pre-write round and the core set $C_r$ that reports this value to $p_r$ replace the step of authenticating the value through a digital signature. This respects safety because $C_r$, for a wise $p_r$, contains at least one correct process that has not altered the value. The full protocol appears in Algorithm 2.
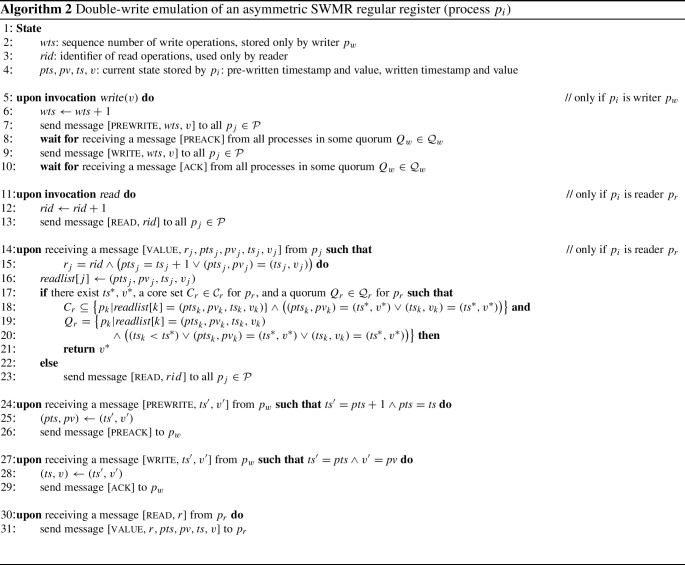


#### Theorem 12

Algorithm 2 emulates an asymmetric Byzantine SWMR regular register, provided there are only finitely many write operations.

#### Proof

We first establish safety when the writer $p_w$ and the reader $p_r$ are wise. In that case, $F \in {\mathcal {F} _w}^* \cap {\mathcal {F} _r}^*$.

During in a *write* operation, $p_w$ has received preack and ack messages from $Q_w \in \mathcal {Q} _i$ and $Q_w' \in \mathcal {Q} _i$, respectively, and for all $Q_r \in \mathcal {Q} _r$ it holds that $Q_w\cap Q_r \not \subseteq F$ and $Q_w'\cap Q_r\not \subseteq F$.

We now argue that any pair $(\textit{ts} ^*,v^*)$ returned by $p_r$ was written by $p_w$ either in a preceding or a concurrent *write*. From the properties of the core set $C_r$, because $p_r$ is wise, and together with the condition that $(\textit{ts} ^*, v^*)$ satisfies, it follows that at least one correct process exists in $C_r$ that stores $(\textit{ts} ^*, v^*)$ as a pre-written or as a written value. Thus, the pair was written by $p_w$ before.

Next we argue that for every completed $\textit{write}(v^*)$ operation, in which $p_w$ has sent $[\textsc {write}, {\textit{wts}, v^*}]$, and for any subsequent *read* operation that selects $(\textit{ts} ^*,v^*)$ and returns $v^*$, it must hold $\textit{wts} \le \textit{ts} ^*$. Namely, the condition on $Q_r$ implies that $\textit{ts} ^* \ge \textit{ts} _k$ for all $p_k \in Q_r$. By the consistency of the quorum system, it holds that $Q_w' \cap Q_r \not \subseteq F$, so there is a correct process $p_\ell \in Q_w'\cap Q_r$ that has sent $\textit{ts} _\ell$ to $p_r$. Then $\textit{ts} ^* \ge \textit{ts} _\ell \ge \textit{wts}$ follows because the timestamp variable of $p_\ell$ only increases.

The combination of the above two paragraphs implies that for *read* operations that are not concurrent with any *write*, the pair $(\textit{ts} ^*,v^*)$ chosen by *read* was actually written in the immediately preceding *write*. If the *read* operation occurs concurrently with a *write*, then the pair $(\textit{ts} ^*,v^*)$ chosen by *read* may also originate from the concurrent *write*. This establishes the safety property of the SWMR regular register.

We now show liveness. First, if $p_w$ is wise, then there exists a quorum $Q_w \in \mathcal {Q} _w$ such that $Q_w \cap F = \emptyset$. Second, any correct process will eventually receive all $[\textsc {prewrite}, {\textit{wts},v}]$ and $[\textsc {write}, {\textit{wts},v}]$ messages sent by $p_w$ and process them in the correct order by the assumption of FIFO links. This means that $p_w$ will receive [preack] and [ack] messages, respectively, from all processes in one of its quorums, since at least the processes in $Q_w$ will eventually send those.

Liveness for the reader $p_r$ is shown under the condition that $p_r$ is wise and that the *read* operation is concurrent with only finitely many *write* operations. The latter condition implies that there is one last *write* operation that is initiated, but does not necessarily terminate, while *read* is active.

By the assumption that $p_w$ is correct and because messages are received in FIFO order, all messages of that last *write* operation will eventually arrive at the correct processes. Notice also that $p_r$ simply repeats its steps until it succeeds and returns a value that fulfills the condition. Hence, there is a time after which all correct processes reply with value messages that contain pre-written and written timestamp/value pairs from that last operation. It is easy to see that there exist a core set and a quorum for $p_r$ that satisfy the condition and the reader returns. In conclusion, the algorithm emulates an asymmetric regular SWMR register, where liveness holds only for finitely many write operations. $\square$

## Broadcast

This section shows how to implement two *broadcast primitives* tolerating Byzantine faults with asymmetric quorums. Recall from the standard literature [28, 30, 32] that reliable broadcasts offer basic forms of reliable message delivery and consistency, but they do not impose a total order on delivered messages (as this is equivalent to consensus). The Byzantine broadcast primitives described here, *consistent broadcast* and *reliable broadcast*, are prominent building blocks for many more advanced protocols.

With both primitives, the sender process may broadcast a message *m* by invoking $\textit{broadcast}(m)$; the broadcast abstraction outputs *m* to the local application on the process through a $\textit{deliver}(m)$ event. Moreover, the notions of broadcast considered in this section are intended to deliver only one message per instance. Every instance has a distinct (implicit) label and a designated sender $p_s$. With standard multiplexing techniques one can extend this to a protocol in which all processes may broadcast messages repeatedly [28].

*Byzantine consistent broadcast* The simplest such primitive, which has been called *(Byzantine) consistent broadcast* [28], ensures only that those correct processes which deliver a message agree on the content of the message, but they may not agree on termination. In other words, the primitive does not enforce “reliability” such that a correct process outputs a message if and only if all other correct processes produce an output. The events in its interface are denoted by *c-broadcast* and *c-deliver*.

The change of the definition towards asymmetric quorums affects most of its guarantees, which hold only for wise processes but not for all correct ones. This is similar to the definition of a register in Sect. 5.

### Definition 9

(*Asymmetric Byzantine consistent broadcast*) A protocol for *asymmetric (Byzantine) consistent broadcast* satisfies:

- *Validity:* If a correct process $p_s$ *c-broadcasts* a message *m*, then all wise processes eventually *c-deliver* *m*.
- *Consistency:* If some wise process *c-delivers* *m* and another wise process *c-delivers* $m'$, then $m=m'$.
- *Integrity:* For any message *m*, every correct process *c-delivers* *m* at most once. Moreover, if the sender $p_s$ is correct and the receiver is wise, then *m* was previously *c-broadcast* by $p_s$.

The following protocol is an extension of “authenticated echo broadcast” [28], which goes back to Srikanth and Toueg [41]. It is a building block found in many Byzantine fault-tolerant protocols with greater complexity. The protocol first has the sender $p_s$ send its message *m* to all processes; then every process echoes *m*, in the sense that it rebroadcasts an echo message with *m* to all processes. As soon as a process receives a quorum of such echo messages that all contain the same $m'$, the process *c-delivers* $m'$. The adaptation for asymmetric quorums is straightforward: Every process considers its own quorum system before *c-delivering* the message.
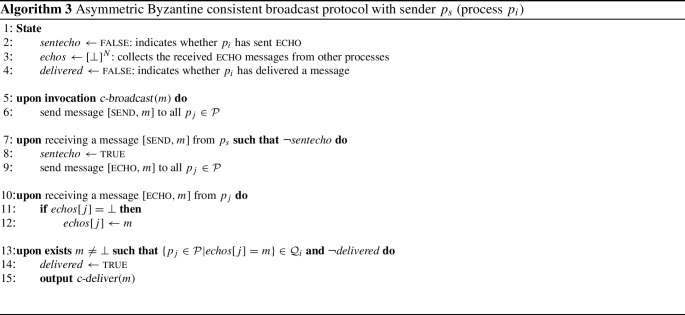


### Theorem 13

Algorithm 3 implements asymmetric Byzantine consistent broadcast.

### Proof

For the *validity* property, it is straightforward to see that every correct process sends $[\textsc {echo}, {m}]$. According to the availability condition for the quorum system $\mathcal {Q} _i$ of every wise process $p_i$ and because $F \subseteq F_i$ for some $F_i \in \mathcal {F} _i$, there exists some quorum $Q_i$ for $p_i$ of correct processes that echo *m* to $p_i$. Hence, $p_i$ *c-delivers* *m*.

To show *consistency*, suppose that some wise process $p_i$ has *c-delivered* $m_i$ because of $[\textsc {echo}, {m_i}]$ messages from a quorum $Q_i$ and another wise $p_j$ has received $[\textsc {echo}, {m_j}]$ from all processes in $Q_j \in \mathcal {Q} _j$. By the consistency property of $\mathbb {Q}$ it holds $Q_i \cap Q_j \not \subseteq F$; let $p_k$ be this process in $Q_i \cap Q_j$ that is not in *F*. Because $p_k$ is correct, $p_i$ and $p_j$ received the same message from $p_k$ and $m_i = m_j$.

The first condition of *integrity* is guaranteed by using the *delivered* flag; the second condition holds because because the receiver is wise, and therefore the quorum that it uses for the decision contains some correct processes that have sent $[\textsc {echo}, {m}]$ with the message *m* they obtained from $p_s$ according to the protocol. $\square$

### Example 4

**Fig. 3:**
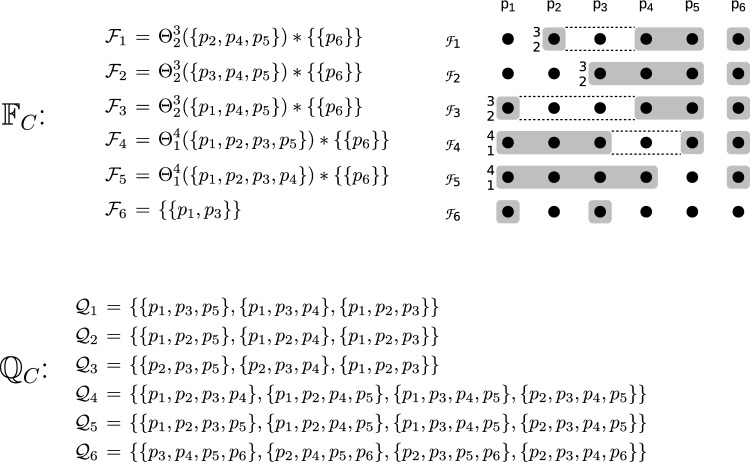
A six-process asymmetric quorum system $\mathbb {Q} _C$, defined through its fail-prone system $\mathbb {F} _C$ and used in Example 4. In $\mathbb {F} _C$, for $p_1$, $p_2$, and $p_3$, each process always trusts itself, some other process of $\{p_1, p_2, p_3\}$ and one further process in $\{p_1, \dots , p_5\}$. Process $p_4$ and $p_5$ each assumes that at most one other process of $\{p_1, \dots , p_5\}$ may fail (excluding itself). Moreover, none of the processes $p_1$, ..., $p_5$ ever trusts $p_6$. For $p_6$ itself, the fail-prone set is $\{p_1, p_3\}$, i.e., it trusts $p_2$, $p_4$, and $p_5$ unconditionally

We illustrate the broadcast protocols using a six-process asymmetric quorum system $\mathbb {Q} _C$, defined through its fail-prone system $\mathbb {F} _C$ and shown in Fig. 3. In $\mathbb {F} _C$, for $p_1$, $p_2$, and $p_3$, each process always trusts itself, some other process of $\{p_1, p_2, p_3\}$ and one further process in $\{p_1, \dots , p_5\}$. Process $p_4$ and $p_5$ each assumes that at most one other process of $\{p_1, \dots , p_5\}$ may fail (excluding itself). Moreover, none of the processes $p_1$, ..., $p_5$ ever trusts $p_6$. For $p_6$ itself, the fail-prone set is $\{p_1, p_3\}$, i.e., it trusts $p_2$, $p_4$, and $p_5$ unconditionally.

One can verify that $B^3(\mathbb {F} _C)$ holds; hence, let $\mathbb {Q} _C$ be the canonical quorum system of $\mathbb {F} _C$. Again, there is no reliable process that could be trusted by all and $\mathbb {Q} _C$ is not a special case of a symmetric threshold Byzantine quorum system. With $F = \{p_1, p_5\}$, for instance, process $p_3$ is wise, $p_2$, $p_4$, and $p_6$ are naïve, and there is no guild.

**Fig. 4:**
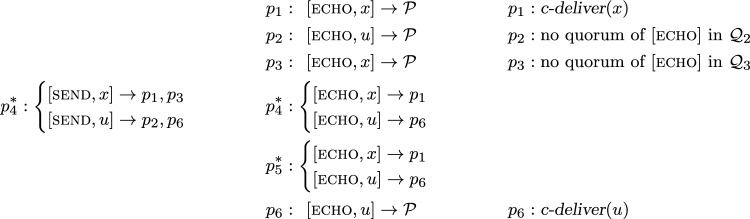
An execution of Algorithm 3 with the asymmetric quorum system $\mathbb {Q} _C$ of Fig. 3, as discussed in Example 4. The sender is $p_4^*$ and processes $F = \{p_4^*, p_5^*\}$ are faulty (the star in $p_4^*$ and $p_5^*$ denotes that they are faulty). This means processes $p_1, p_2, p_3$ are wise and form a guild because $\{p_1, p_2, p_3 \}$ is a quorum for all three; furthermore, $p_6$ is naïve. The wise process $p_1$ *c-delivers* *x* and the naïve process $p_6$ *c-delivers* *u*

Consider now an execution of Algorithm 3 with sender $p_4^*$ and $F = \{p_4^*, p_5^*\}$ (we write $p_4^*$ and $p_5^*$ to denote that they are faulty). This means processes $p_1, p_2, p_3$ are wise and form a guild because $\{p_1, p_2, p_3 \}$ is a quorum for all three; furthermore, $p_6$ is naïve. A protocol execution may proceed as shown in Fig. 4.

Process $p_1$ receives $[\textsc {echo}, {x}]$ from, say, $\{p_1, p_3, p_4^*\} \in \mathcal {Q} _1$ and *c-delivers* *x*, but the other wise processes do not terminate. The naïve $p_6$ gets $[\textsc {echo}, {u}]$ from $\{p_2, p_4^*, p_5^*, p_6\} \in \mathcal {Q} _6$ and *c-delivers* $u \ne x$.

*Byzantine reliable broadcast* In the symmetric setting, consistent broadcast has been extended to *(Byzantine) reliable broadcast* in a well-known way to address the disagreement about termination among the correct processes [28]. This primitive has the same interface as consistent broadcast, except that its events are called *r-broadcast* and *r-deliver* instead of *c-broadcast* and *c-deliver*, respectively.

A reliable broadcast protocol also has all properties of consistent broadcast, but satisfies the additional *totality* property stated next. Taken together, *consistency* and *totality* imply a notion of *agreement*, similar to what is also ensured by many crash-tolerant broadcast primitives. Analogously to the earlier primitives with asymmetric trust, our notion of an *asymmetric reliable broadcast*, defined next, ensures agreement on termination only for the wise processes, and moreover only for executions with a guild. Also the *validity* of Definition 9 is extended by the assumption of a guild. Intuitively, one needs a guild because the wise processes that make up the guild are self-sufficient, in the sense that the guild contains a quorum of wise processes for each of its members; without that, there may not be enough wise processes.

### Definition 10

(*Asymmetric Byzantine reliable broadcast*) A protocol for *asymmetric (Byzantine) reliable broadcast* is a protocol for asymmetric Byzantine consistent broadcast with the revised *validity* condition and the additional *totality* condition stated next:

- *Validity:* In all executions with a guild, if a correct process $p_s$ *r-broadcasts* a message *m*, then all processes in the maximal guild eventually *r-deliver* *m*.
- *Totality:* In all executions with a guild, if a wise process *r-delivers* some message, then all processes in the maximal guild eventually *r-deliver* a message.

The protocol of Bracha [21] implements reliable broadcast subject to Byzantine faults with symmetric trust. It augments the authenticated echo broadcast from Algorithm 3 with a second all-to-all exchange, where each process is supposed to send ready with the payload message that will be *r-delivered*. When a process receives the same *m* in $2f+1$ ready messages, in the symmetric model with a threshold Byzantine quorum system, then it *r-delivers* *m*. Also, a process that receives $[\textsc {ready}, {m}]$ from $f+1$ distinct processes and that has not yet sent a ready chimes in and also sends $[\textsc {ready}, {m}]$. These two steps ensure totality.

For asymmetric quorums, the conditions of a process $p_i$ receiving $f+1$ and $2f+1$ equal ready messages, respectively, generalize to receiving the same message from a kernel for $p_i$ and from a quorum for $p_i$. Intuitively, the change in the first condition ensures that when a wise process $p_i$ (that is also in the maximal guild) receives the same $[\textsc {ready}, {m}]$ message from a kernel for itself, then this kernel intersects with some quorum of wise processes. Therefore, at least one wise process has sent $[\textsc {ready}, {m}]$ and $p_i$ can safely adopt *m*. Furthermore, the change in the second condition relies on the properties of asymmetric quorums to guarantee that whenever some wise process has *r-delivered* *m*, then enough correct processes have sent a $[\textsc {ready}, {m}]$ message such that all wise processes eventually receive a kernel of $[\textsc {ready}, {m}]$ messages and also send $[\textsc {ready}, {m}]$.
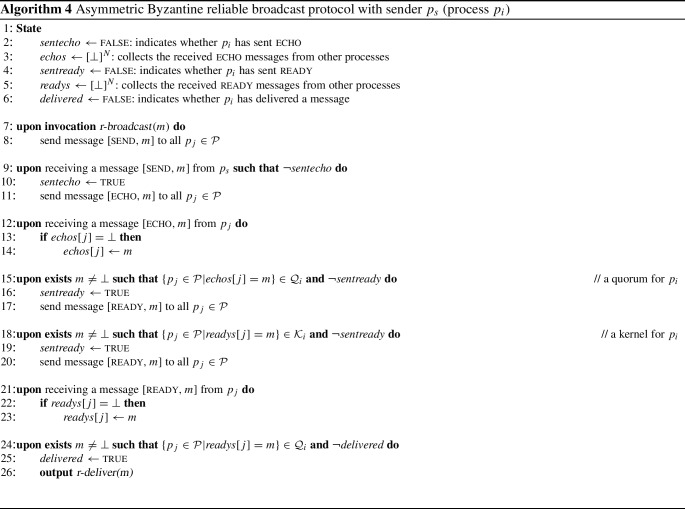


Applying these changes to Bracha’s protocol results in the asymmetric reliable broadcast protocol shown in Algorithm 4. Note that it strictly extends Algorithm 3 by the additional round of ready messages, in the same way as for symmetric trust. For instance, when instantiated with the symmetric threshold quorum system of $n = 3f + 1$ processes, of which *f* may fail, then every set of $f + 1$ processes is a kernel.

In Algorithm 4, there are two conditions that let a correct $p_i$ send $[\textsc {ready}, {m}]$: either receiving a quorum of $[\textsc {echo}, {m}]$ messages for itself or obtaining a kernel for itself of $[\textsc {ready}, {m}]$ messages. For the first case, we say $p_i$ *sends* ready *after* echo ; for the second case, we say $p_i$ *sends* ready *after* ready .

### Lemma 14

In any execution with a guild, there exists a unique *m* such that whenever a wise process in the maximal guild sends a ready message, it contains *m*.

### Proof

Consider first all ready messages sent by wise processes after echo. The fact that Algorithm 4 extends Algorithm 3 achieving consistent broadcast, combined with the consistency property in Definition 9 implies immediately that the lemma holds for ready messages sent by wise processes after echo.

For the second case, let $\mathcal {G} _{\text {max}}$ be the maximal guild. Consider the first wise process $p_i$ in $\mathcal {G} _{\text {max}}$ which sends $[\textsc {ready}, {m'}]$ after ready. From the protocol it follows that all processes in some kernel $K_i \in \mathcal {K} _i$, which triggered $p_i$ to send $[\textsc {ready}, {m'}]$, have sent $[\textsc {ready}, {m'}]$ to $p_i$. Moreover, according to the definition of a kernel, $K_i$ overlaps with all quorums for $p_i$. Since $p_i$ is in the (maximal) guild, at least one of the quorums for $p_i$ consists exclusively of wise processes. Hence, some wise process $p_j$ in the guild has sent $[\textsc {ready}, {m'}]$ to $p_i$. But since $p_i$ is the first wise process to send ready after ready, it follows that $p_j$ sent $[\textsc {ready}, {m'}]$ after echo; therefore, $m' = m$ from the proof in the first case. Continuing this argument inductively over all ready messages sent after ready by wise processes in $\mathcal {G} _{\text {max}}$, in the order these were sent, shows that all those messages contain *m* and establishes the lemma. $\square$

### Theorem 15

Algorithm 4 implements asymmetric Byzantine reliable broadcast.

### Proof

Recall that the *validity* property assumes there exists a maximal guild $\mathcal {G} _{\text {max}}$. Since the sender $p_s$ is correct and according to asymmetric quorum availability, every process $p_i$ in $\mathcal {G} _{\text {max}}$ eventually receives a quorum of $[\textsc {echo}, {m}]$ messages for itself, containing the message *m* from $p_s$. According to the protocol, $p_i$ therefore sends $[\textsc {ready}, {m}]$ after echo unless $\textit{sentready} = \textsc {true}$; if this is the case, however, $p_i$ has already sent $[\textsc {ready}, {m}]$ after ready as ensured by Lemma 14. Hence, every process in $\mathcal {G} _{\text {max}}$ eventually sends $[\textsc {ready}, {m}]$. Then every process $p_j$ in $\mathcal {G} _{\text {max}}$ eventually receives a quorum for itself of $[\textsc {ready}, {m}]$ messages and *r-delivers* *m*, as ensured by the properties of a guild and by the protocol.

To establish the *totality* condition, suppose that some wise process $p_i$ has *r-delivered* a message *m*. Then it has obtained $[\textsc {ready}, {m}]$ messages from the processes in some quorum $Q_i \in \mathcal {Q} _i$.

Consider any other wise process $p_j \in \mathcal {G} _{\text {max}}$. Since $p_i$ and $p_j$ are both wise, it holds $F \in {\mathcal {F} _i}^*$ and $F \in {\mathcal {F} _j}^*$, which implies $F \in {\mathcal {F} _i}^* \cap {\mathcal {F} _j}^*$. Then, the set $K = Q_i \setminus F$ intersects every quorum of $p_j$ by quorum consistency and therefore contains a kernel for $p_j$. Since *K* consists only of correct processes, all of them have sent $[\textsc {ready}, {m}]$ also to $p_j$ and $p_j$ eventually sends $[\textsc {ready}, {m}]$ as well. This implies that all wise processes in $\mathcal {G} _{\text {max}}$ eventually send $[\textsc {ready}, {m}]$ to all processes. With the same argument as just given for validity, it follows that every wise process in the guild receives a quorum for itself of $[\textsc {ready}, {m}]$ and *r-delivers* *m*, as required for totality.

The *consistency* property follows immediately from the preceding argument and from Lemma 14, which implies that all wise processes deliver the same message.

Finally, *integrity* holds because of the *delivered* flag in the protocol and because of the argument showing validity together with Lemma 14. $\square$

### Example 5

**Fig. 5:**
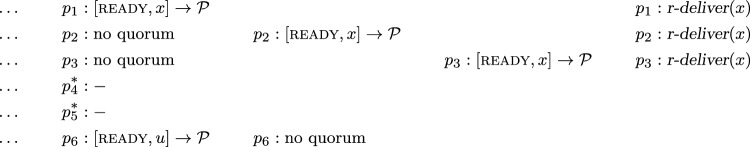
An execution of Algorithm 4 with the asymmetric quorum system $\mathbb {Q} _C$ of Fig. 3, as discussed in Example 5. The figure shows how the execution continues after the steps shown in Fig. 4. The set $\{p_1, p_2, p_3\}$ is the maximal guild and contains a quorum for each of its members, hence all three wise processes *r-deliver* *x*

Consider again the protocol execution with quorum system $\mathbb {Q} _{C}$ shown in Fig. 3 and introduced in Example 4. Recall the execution of asymmetric consistent broadcast from Fig. 4 and observe that with $F = \{p_4^*, p_5^*\}$, the set $\{p_1, p_2, p_3\}$ is a guild and $p_6$ is naïve. The start of the execution is the same as shown previously and omitted here. Instead of *c-delivering* *x* and *u*, respectively, $p_1$ and $p_6$ send $[\textsc {ready}, {x}]$ and $[\textsc {ready}, {u}]$ to all processes. Figure 5 shows how this execution continues.

Note that the kernel systems of processes $p_1$, $p_2$, and $p_3$ are, respectively, $\mathcal {K} _1 = \{\{p_1\}, \{p_3\}\}$, $\mathcal {K} _2 = \{\{p_1\}, \{p_2\}\}$, and $\mathcal {K} _3 = \{\{p_2\}, \{p_3\}\}$. Hence, when $p_2$ receives $[\textsc {ready}, {x}]$ from $p_1$, it sends $[\textsc {ready}, {x}]$ in turn because $\{ p_1 \}$ is a kernel for $p_2$, and when $p_3$ receives this message, then it sends $[\textsc {ready}, {x}]$ because $\{ p_2 \}$ is a kernel for $p_3$.

Furthermore, since $\{p_1, p_2, p_3\}$ is the maximal guild and contains a quorum for each of its members, all three wise processes *r-deliver* *x* as implied by *consistency* and *totality*. The naïve $p_6$ does not *r-deliver* anything, however.

*Remarks* Asymmetric reliable broadcast (Definition 10) ensures validity and totality only for processes in the maximal guild. There may exist wise processes outside the maximal guild that do not terminate. On the other hand, asymmetric consistent broadcast (Definition 9) ensures validity also for all *wise* processes.

Another open questions concerns the conditions for reacting to ready messages in the asymmetric reliable broadcast protocol. Already in Bracha’s protocol for the threshold model [21], a process (1) sends its own ready message upon receiving $f+1$ ready messages and (2) *r-delivers* an output upon receiving $2f+1$ ready messages. These conditions generalize for arbitrary, non-threshold quorum systems to receiving messages (1) from any set that is guaranteed to contain at least one correct process and (2) from any set that still contains at least one process even when any two fail-prone process sets are subtracted. In Algorithm 4, in contrast, a process delivers the payload only after receiving ready messages from one of its quorums. But such a quorum (e.g., $\big \lceil \frac{n+f+1}{2}\big \rceil$ processes) may be larger than a set in the second case (e.g., $2f+1$ processes). It remains interesting to find out whether this discrepancy is necessary.

## Consensus

In this section we define asymmetric asynchronous Byzantine consensus and implement it through a randomized algorithm, which extends and improves the protocol of Mostéfaoui et al. [4].

The protocol of Mostéfaoui et al. comes in multiple versions. The original one, published at PODC 2014 [4] and where it also won the best-paper award, suffers from a subtle and little-known liveness problem [27]: an adversary can prevent progress among the correct processes by controlling the messages between them and by sending them values in a specific order. The subsequent version (JACM 2015) [5] resolves this issue, but requires many more communication steps and adds considerable complexity.

In Appendix A we show in detail how it is possible to violate liveness in the PODC 2014 version. We also propose a method that overcomes the problem, maintains the elegance of the protocol, and does not affect its appealing properties. Based on this insight, in this section, we show how to realize asynchronous consensus with asymmetric trust, again with a protocol that maintains the simplicity of the original approach of Mostéfaoui et al. [4].

### Definition

In an asynchronous binary consensus protocol, every correct process initially *ac-proposes* a bit; the protocol concludes at a correct process when it *ac-decides* a bit. Our notion of Byzantine consensus uses strong validity in the asymmetric model. Furthermore, it restricts the safety properties of consensus from all correct ones to *wise* processes in the guild. For implementing asynchronous consensus, we use a system enriched with randomization. In round-based consensus algorithms, the termination property is formulated with respect to the round number *r* that a process executes. The corresponding probabilistic asymmetric termination property is guaranteed only for wise processes in the maximal guild.

#### Definition 11

(*Asymmetric strong Byzantine consensus*) A protocol for asynchronous *asymmetric strong Byzantine consensus* satisfies:

- *Probabilistic termination:* In all executions with a non-empty guild, every process in the maximal guild $\textit{ac-decides}$ with probability 1, i.e., for all $p_i \in \mathcal {G} _{\text {max}}$,
  $$\begin{aligned} \lim _{r \rightarrow + \infty } (\textrm{P}[\text {process }p_i \textit{ac-decides}\text { by round }r]) = 1. \end{aligned}$$
  *Strong validity:* In all executions with a non-empty guild, a wise process only $\textit{ac-decides}$ a value that has been $\textit{ac-proposed}$ by some process in the maximal guild.
- *Integrity:* No correct process $\textit{ac-decides}$ twice.
- *Agreement:* No two wise processes $\textit{ac-decide}$ differently.

The consensus protocol described here relies in a modular way on two subprotocols. Recall from Sect. 3 that all processes are connected pairwise by reliable FIFO links. The FIFO guarantees on the links hold across multiple protocol modules.

### Asymmetric common coin

Our randomized consensus algorithm delegates its probabilistic choices to a *common coin* abstraction [24, 28]. This primitive is triggered by a *release-coin* invocation and terminates by generating an *output-coin*(*s*) event, where $s \in \mathcal {B}$ represents the random coin value in a range $\mathcal {B}$. We define this in the asymmetric-trust model. The coin remains hidden and unpredictable by faulty processes up to the time when sufficiently many wise processes have released it. This is the case when at least a set of correct processes that is a kernel for all wise processes have released it.

#### Definition 12

(*Asymmetric common coin*) A protocol for *asymmetric common coin* satisfies the following properties:

- *Termination:* In all executions with a non-empty guild, every process in the maximal guild eventually outputs a coin value.
- *Unpredictability:* In all executions with a non-empty guild, no process has any information about the value of the coin before at least a kernel for all wise processes, which consists entirely of correct processes, has released the coin.
- *Matching:* In all executions with a guild, with probability 1 every process in the maximal guild outputs the same coin value.
- *No bias:* The distribution of the coin is uniform over $\mathcal {B}$.

Here we consider binary consensus and $\mathcal {B} = \{0,1\}$. The *termination* property guarantees that every process in the maximal guild eventually outputs a coin value that is ensured to be the same for each of them by the *matching* property. The *unpredictability* property ensures that the coin value is kept secret in an execution until at least a kernel for a wise process, consisting entirely of wise processes, releases the coin. The existence of a kernel with only *wise* processes is required in order to avoid a liveness problem in the consensus protocol (we describe this in Appendix A). The analogue of this in the threshold symmetric model, where $f < n / 3$ processes may fail, would be a coin with threshold 2*f*, where the value is kept secret until at least a set of $f+1$ *correct* processes have released the coin. Finally, the *no bias* property specifies the probability distribution of the coin output.

*The scheme* We recall here the notion of the *tolerated system* of an asymmetric Byzantine quorum system from Sect. 4.2. Every asymmetric Byzantine quorum system $\mathbb {Q}$ induces a tolerated system $\mathcal {T}$ that contains sets *T* that are the complement of the maximal guild in some execution, i.e., $T = \mathcal {P} {\setminus } \mathcal {G} _{\max }$ and $\mathcal {G} _{\max }$ is a maximal guild for some execution and for $\mathbb {Q}$. Crucial for our application is the fact that $\mathcal {T}$ satisfies the $Q^3$-condition (Lemma 10), hence one can construct a *symmetric* Byzantine quorum system from $\mathcal {T}$. In particular, the corresponding canonical system $\mathcal {H}$ containing all possible maximal guilds, is such a symmetric Byzantine quorum system. The idea is to use the tolerated system as a “bridge” from the asymmetric to the symmetric model, since reasoning is simpler in the latter. At the same time, this approach guarantees that in any execution where the system is able to make progress because a non-empty guild exists, the protocol can exploit the fact that such a guild exists also for a safety property.

The common coin scheme follows the approach of Rabin [24] and assumes that coins are predistributed by a trusted dealer. The scheme uses Benaloh-Leichter [42] secret sharing, such that the coin is additively shared within every maximal guild. The dealer shares one coin for every possible round of the protocol. This requires knowledge of the symmetric Byzantine quorum system $\mathcal {H}$ corresponding to the tolerated system $\mathcal {T}$. Observe that every process can compute this because $\mathbb {F}$ is globally known.

We assume that before the coin protocol runs, the dealer has chosen uniformly at random a value $s \in \mathcal {B}$ and shared it as follows. For every possible maximal guild $\mathcal {G} = \{ p_{i_1}, \dots , p_{i_{m}} \}$ across all executions, the dealer has picked uniform shares $s_{i_1}^\mathcal {G}, \dots , s_{i_{m-1}}^\mathcal {G}$ and set $s_{i_m}^\mathcal {G} = s + \sum _{\ell =1}^{m-1} s_{i_\ell }^\mathcal {G}$. Then the dealer has given share $s_{i_\ell }^\mathcal {G}$ to process $p_{i_\ell }$, for $\ell \in \{1,\dots ,m\}$. This implies that process $p_i$ holds a share for every guild of which it is a member.

The code for process $p_i$ to release the coin is shown in Algorithm 5. Specifically, when asked to release its coin share (Lines 4–8, Algorithm 5), a process $p_i$ sends to all other processes a share $s_\mathcal {G}$ for each guild $\mathcal {G}$ of which $p_i$ is a member. Upon receiving such shares, each process stores them in a local structure (Lines 9–11, Algorithm 5). When a process $p_i$ has enough shares, i.e., all shares from a guild $\mathcal {G}$, it can locally add them and output the coin value (Lines 12–14, Algorithm 5).
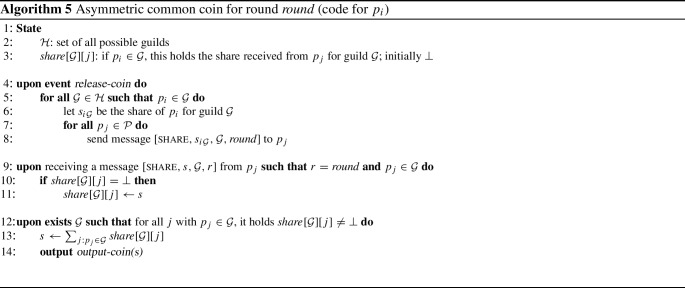


#### Theorem 16

Algorithm 5 implements an asymmetric common coin.

#### Proof

Let us consider an asymmetric fail-prone system $\mathbb {F}$ such that $B^3(\mathbb {F})$ holds and the corresponding asymmetric Byzantine quorum system $\mathbb {Q}$ for $\mathbb {F}$. By Lemma 10, the tolerated system $\mathcal {T}$ of $\mathbb {Q}$ satisfies the $Q^3$-condition. Let $\mathcal {H}$ be the Byzantine quorum system for $\mathcal {T}$ consisting of all maximal guilds. Assume an execution with a guild, where all processes in some $F \in \mathcal {T}^*$ are faulty and $\mathcal {G} \in \mathcal {H}$ is the maximal guild.

For the *termination* property, observe that every correct process, and hence also every process in $\mathcal {G}$, invokes $\textit{release-coin}$. This implies that every process $p_i \in \mathcal {G}$ sends share messages to all processes in $\mathcal {P}$ (Line 8, Algorithm 5) containing the coin shares of $p_i$ for every guild in which $p_i$ belongs (Line 5, Algorithm 5), including $\mathcal {G}$. Eventually every correct process in $\mathcal {P}$ receives a share message from every process in $\mathcal {G}$, computes *s* (Line 13, Algorithm 5) and triggers $\textit{output-coin}(s)$. We note that termination holds actually for all correct processes, not just for those in the maximal guild.

For the *unpredictability* property, assume a correct process $p_i$ outputs coin *s*. This implies the existence of a set $\mathcal {G}_k \in \mathcal {H}$, where each member of $\mathcal {G}_k$ has sent a share message. Now, let us define *K* as the set $\mathcal {G}_k {\setminus } F$. Observe that by construction, *K* always contains a process $p_i$ in $\mathcal {G}$ that is wise, since $\mathcal {G}$ is the maximal guild in the execution. This is a consequence of $\mathcal {H}$ being a Byzantine quorum system, ensuring $\mathcal {G}_k \cap \mathcal {G} \not \subseteq F$. This also implies that $K = \mathcal {G}_k {\setminus } F \cap \mathcal {G} \ne \emptyset$.

We first prove that this *K* intersects with every quorum of every wise process in the execution.

Suppose by contradiction that there exists a wise process $p_j \in \mathcal {P}$ with a quorum $Q_j \in \mathcal {Q}_j$ such that $Q_j \cap K = \emptyset$. Let $p_i$ be a process in $K \cap \mathcal {G}$. Given $K \subseteq \mathcal {G}_k$ and that $\mathcal {G}_k$ is a guild within $\mathcal {H}$, there must exist a quorum $Q_i \in \mathcal {Q}_i$ for $p_i$ such that $Q_i \subseteq \mathcal {G}_k$. However, if $Q_j \cap K = \emptyset$ and $K = \mathcal {G}_k \setminus F$, it follows that $Q_i \cap Q_j \subseteq F$. This situation contradicts the consistency property of the quorum system $\mathbb {Q}$.

Furthermore, employing the reasoning used in Lemma 3, we can derive from *K* a minimal set that continues to intersect with every quorum of every wise process. Therefore, it follows that *K* contains a kernel for every wise process, consisting only of correct processes.

The *matching* and *no bias* properties follow directly from the fact that the coin value for every round is predetermined, albeit not known to any process, and chosen uniformly at random by the trusted dealer. $\square$

#### Example 6

**Fig. 6:**
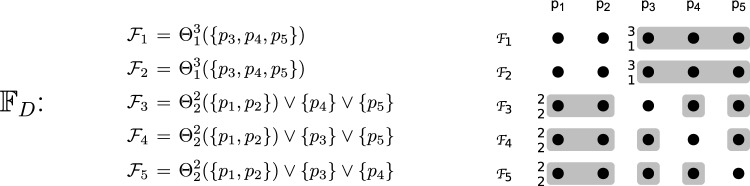
The asymmetric fail-prone system $\mathbb {F} _D$ with five processes described in Example 6

Let us consider a five-process asymmetric quorum system $\mathbb {Q} _D$, defined through $\mathbb {F} _D$ shown in Fig. 6.

The tolerated system is $\mathcal {T} = \{\{p_1, p_2\}, \{p_3\}, \{p_4\}, \{p_5\}\}$. One can verify that $B^3(\mathbb {F} _D)$ holds; hence, by Lemma 10, also $Q^3(\mathcal {T})$ holds. The corresponding symmetric Byzantine quorum system is

$$\begin{aligned} \mathcal {H}&= \{\{p_3, p_4, p_5\}, \{p_1, p_2, p_4 , p_5\}, \\&\quad \{p_1, p_2, p_3 , p_5\}, \{p_1, p_2, p_3 , p_4\}\}. \end{aligned}$$

Observe that every $\mathcal {G} \in \mathcal {H}$ is a guild in an execution in which the processes in $T = \mathcal {P} \setminus \mathcal {G}$ are faulty.

Let us assume an execution with a set of faulty processes $F=\{p_1,p_2\}$; this implies that the guild in this execution is $\{p_3,p_4,p_5\}$. We show how Algorithm 5 works.

Let us assume that the dealer has chosen $s=1$. Then, for every guild $\mathcal {G} _k\in \mathcal {H}$, with $k \in \{1,\ldots ,4\}$, the dealer has chosen uniform shares as follows, where $s^k_i$ denotes the share of process *i* for guild $\mathcal {G} _k$.

$$\begin{aligned} \mathcal {G} _1&= \{p_3, p_4, p_5\}:\\&s^1_3=1, s^1_4=0 \text {, and } s^1_5 = s + s^1_3 + s^1_4 = 0 \\ \mathcal {G} _2&= \{p_1, p_2, p_4 , p_5\}:\\&s^2_1=0, s^2_2=1, s^2_4=1 \text {, and } s^2_5 = s + s^2_1 + s^2_2 + s^2_4 = 1 \\ \mathcal {G} _3&= \{p_1, p_2, p_3 , p_5\}:\\&s^3_{1}=0, s^3_{2}=1, s^3_{3}=0 \text {, and } s^3_{5} = s + s^3_{1} + s^3_{2} + s^3_{3} = 0 \\ \mathcal {G} _4&= \{p_1, p_2, p_3 , p_4\}:\\&s^4_{1}=1, s^4_{2}=0, s^4_{3}=0 \text {, and } s^4_{4} = s + s^4_{1} + s^4_{2} + s^4_{3} = 0 \end{aligned}$$

Every process in $\mathcal {G} _1=\{p_3,p_4,p_5\}$ upon *release-coin* sends a share message to every process $p_j \in \mathcal {P}$ for every share it has.

Process $p_3$ is part of $\mathcal {G} _1, \mathcal {G} _3$ and $\mathcal {G} _4$. This means that upon *release-coin*, $p_3$ sends $[\textsc {share}, {1, 1, 1}]$, $[\textsc {share}, {0, 3, 1}]$ and $[\textsc {share}, {0, 4, 1}]$ to every process in $\mathcal {P}.$

Process $p_4$ is part of $\mathcal {G} _1, \mathcal {G} _2$ and $\mathcal {G} _4$. This means that upon *release-coin*, $p_4$ sends $[\textsc {share}, {0, 1, 1}]$, $[\textsc {share}, {1, 2, 1}]$ and $[\textsc {share}, {0, 4, 1}]$ to every process in $\mathcal {P}.$

Process $p_5$ is part of $\mathcal {G} _1, \mathcal {G} _2$ and $\mathcal {G} _3$. This means that upon *release-coin*, $p_5$ sends $[\textsc {share}, {0, 1, 1}]$, $[\textsc {share}, {1, 2, 1}]$ and $[\textsc {share}, {0, 3, 1}]$ to every process in $\mathcal {P}.$

Eventually every process in $\mathcal {G} _1$ receives a share message of the form $[\textsc {share}, {s^1_i, 1, 1}]$ from each process $p_i \in \mathcal {G} _1$, computes $s \leftarrow \sum _{i: p_i \in \mathcal {G} _1} s^1_i$ (Line 13, Algorithm 5) and $\textit{output-coin}(1)$.

*Discussion* This implementation is expensive because the number of shares for one particular coin held by a process $p_i$ is equal to the number of guilds in which $p_i$ is contained. It would be more efficient to implement an asymmetric coin “from scratch” according to the protocols of Canetti and Rabin [43] or of Patra et al. [44]. Alternatively, distributed cryptographic implementations are possible, for example, implementations relying on the hardness of the discrete logarithm problem [45].

### Asymmetric binary validated broadcast

We generalize the binary validated broadcast as introduced by Mostéfaoui et al. [4] and as reviewed in Appendix A.1 to the asymmetric-trust model. In this primitive, every process may broadcast a bit $b \in \{0,1\}$ by invoking $\textit{abv-broadcast}(b)$. The primitive outputs at least one binary value and possibly also both binary values through an *abv-deliver* event. This means one or two *abv-deliver* events might occur at a correct process, which separates this notion from the broadcasts of the previous section. In the asymmetric version, all safety properties are restricted to wise processes, and a guild is required for liveness. This gives the following notion.

#### Definition 13

(*Asymmetric binary validated broadcast*) A protocol for *asymmetric binary validated broadcast* satisfies the following properties:

- *Validity:* In all executions with a guild, let *K* be a kernel for every process in the maximal guild. If every process in *K* is correct and has *abv-broadcast* the same value $b \in \{0,1\}$, then every wise process eventually *abv-delivers* *b*.
- *Integrity:* In all executions with a guild, if a wise process *abv-delivers* some *b*, then *b* has been *abv-broadcast* by some process in the maximal guild.
- *Agreement:* In all executions with a guild, if a wise process *abv-delivers* some value *b*, then every wise process eventually *abv-delivers* *b*.
- *Termination:* In all executions with a guild, every wise process eventually *abv-delivers* some value.

Note that it guarantees properties only for processes that are wise. Liveness properties also assume there exists a guild.
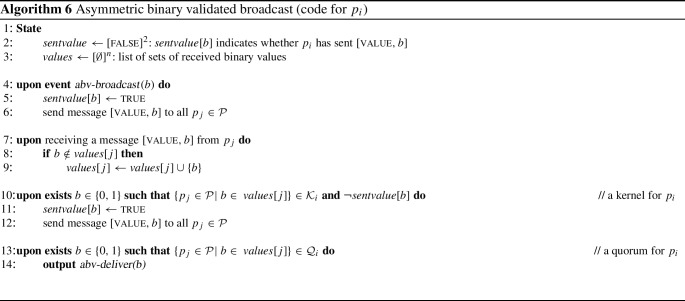


Algorithm 6 works in the same way as the binary validated broadcast by Mostéfaoui et al. [4], but differs in the use of an asymmetric quorum and kernel systems. When a correct process $p_i$ invokes $\textit{abv-broadcast}(b)$ for $b \in \{0,1\}$, it sends a value message containing *b* to all processes. Afterwards, whenever a correct process $p_i$ receives value messages containing *b* from from a kernel $K_i$ for itself and has not sent a value message containing *b* itself, then it sends such message to every process. Finally, once a correct process $p_i$ receives value messages containing *b* from a quorum $Q_i$ for itself, it delivers *b* through $\textit{abv-deliver}(b)$. Note that a process *abv-delivers* at least one and at most two values.

#### Theorem 17

Algorithm 6 implements asymmetric binary validated broadcast.

#### Proof

To prove the *validity* property, let us consider a kernel *K* for every process $p_i$ in the maximal guild $\mathcal {G} _{\text {max}}$. Moreover, let us assume that every process in *K* has $\textit{abv-broadcast}$ the same value $b \in \{0,1\}$. Then, by definition of a kernel, *K* intersects every $Q_i$ for every $p_i \in \mathcal {G} _{\text {max}}$. According to the protocol, every process in $\mathcal {G} _{\text {max}}$ eventually sends $[\textsc {value}, {b}]$ unless $\textit{sentvalue}[b] = \textsc {true}$ for some $p_i \in \mathcal {G} _{\text {max}}$. However, if $\textit{sentvalue}[b] = \textsc {true}$ for $p_i$, process $p_i$ has already sent $[\textsc {value}, {b}]$. Since every process in the maximal guild eventually sends $[\textsc {value}, {b}]$, eventually every correct process $p_j$ also receives $[\textsc {value}, {b}]$ from a kernel for itself (see Corollary 8) and sends $[\textsc {value}, {b}]$ unless $\textit{sentvalue}[b] = \textsc {true}$. However, as above, if $\textit{sentvalue}[b] = \textsc {true}$ for $p_j$, process $p_j$ has already sent $[\textsc {value}, {b}]$. It follows that eventually every wise process receives a quorum for itself of values *b* and *abv-delivers* *b*.

For the *integrity* property, let us assume an execution with a maximal guild $\mathcal {G} _{\text {max}}$. Suppose first that only Byzantine processes *abv-broadcast* *b*. Then, the set consisting of only these processes cannot form a kernel for any wise process. It follows that Line 10 of Algorithm 6 cannot be satisfied. If only naïve processes *abv-broadcast* *b*, then by the definition of a quorum system and by the assumed existence of a maximal guild, there is at least one quorum for every process in $\mathcal {G} _{\text {max}}$ that does not contain any naïve processes (e.g., as in Example 2). All naïve processes together cannot be a kernel for processes in $\mathcal {G} _{\text {max}}$. Again, Line 10 of Algorithm 6 cannot be satisfied. Finally, let us assume that a wise process $p_i$ outside the maximal guild *abv-broadcasts* *b*. Then, $p_i$ cannot be a kernel for every wise process: it is not part of the quorums inside $\mathcal {G} _{\text {max}}$. It follows that if a wise process *abv-delivers* some *b*, then *b* has been *abv-broadcast* by some processes in the maximal guild.

To show *agreement*, let *F* be the set of faulty processes and suppose that a wise process $p_i$ has *abv-delivered* *b*. Then it has obtained $[\textsc {value}, {b}]$ messages from the processes in some quorum $Q_i \in \mathcal {Q} _i$ and before from a kernel $K=Q_i \setminus F$ for itself. Each correct process in *K* has sent $[\textsc {value}, {b}]$ message to all other processes. Consider any other wise process $p_j$. Since $p_i$ and $p_j$ are both wise, we have $F \in {\mathcal {F} _i}^*$ and $F \in {\mathcal {F} _j}^*$, which implies $F \in {\mathcal {F} _i}^* \cap {\mathcal {F} _j}^*$. It follows that *K* is also a kernel for $p_j$. Thus, $p_j$ sends a $[\textsc {value}, {b}]$ message to every process. This implies that all wise processes eventually send $[\textsc {value}, {b}]$ to all processes. This also implies that eventually every process in $\mathcal {G} _{\text {max}}$ sends $[\textsc {value}, {b}]$. By Corollary 8, $\mathcal {G} _{\text {max}}$ contains a kernel for every correct process $p_k$. Thus, $p_k$ sends a $[\textsc {value}, {b}]$ message to every process. Therefore eventually every wise process receives a quorum for itself of $[\textsc {value}, {b}]$ messages and *abv-deliver* *b*.

For the *termination* property, let us assume an execution with a maximal guild $\mathcal {G} _{\text {max}}$ and set of faulty processes *F*. Note that in any execution, every process in $\mathcal {P} {\setminus } F$ *abv-broadcasts* some binary values. We show that there is a set $K \subseteq \mathcal {P} \setminus F$ such that *K* is a kernel for every process in the maximal guild consisting of correct processes and every process in *K* *abv-broadcasts* the same value $b \in \{0,1\}$. Observe that a correct process initially *abv-broadcasts* only one value in $\{0,1\}$. So, let $\mathcal {P} {\setminus } F = S_0 \cup S_1$ with $S_0$ and $S_1$ two sets of processes such that $S_0 \cap S_1 = \emptyset$ and such that every process in $S_0$ *abv-broadcasts* *b* and every process in $S_1$ *abv-broadcasts* $1-b = \overline{b}$. Moreover, let us assume that neither $S_0$ nor $S_1$ contains a kernel for every process in the maximal guild. If $S_0$ does not contain a kernel for a process in the maximal guild, then there exists a process $p_j \in \mathcal {G} _{\text {max}}$ and a quorum $Q_j$ for $p_j$ such that $Q_j \cap S_0 = \emptyset$. This means that every correct process in $Q_j$ *abv-broadcasts* $\overline{b}$. Similarly, if $S_1$ does not contain a kernel for a process in the maximal guild, then there exists a process $p_k \in \mathcal {G} _{\text {max}}$ and a quorum $Q_k$ for $p_k$ such that $Q_k \cap S_1 = \emptyset .$ This means that every correct process in $Q_k$ *abv-broadcasts* *b*. However, if this is the case, then $Q_j \cap Q_k \subseteq F$, which contradicts the consistency property of an asymmetric Byzantine quorum system, given that $p_j$ and $p_k$ are both wise. This implies that either $S_0$ or $S_1$ contains a kernel *K* for every process in the maximal guild consisting of correct processes and such that every process in *K* *abv-broadcasts* the same value. Termination then follows from the validity property. $\square$

### Asymmetric randomized consensus

In consensus, a correct process may *propose* a binary value *b* by invoking $\textit{ac-propose}(b)$, and the consensus abstraction *decides* for *b* through an $\textit{ac-decide}(b)$ event.
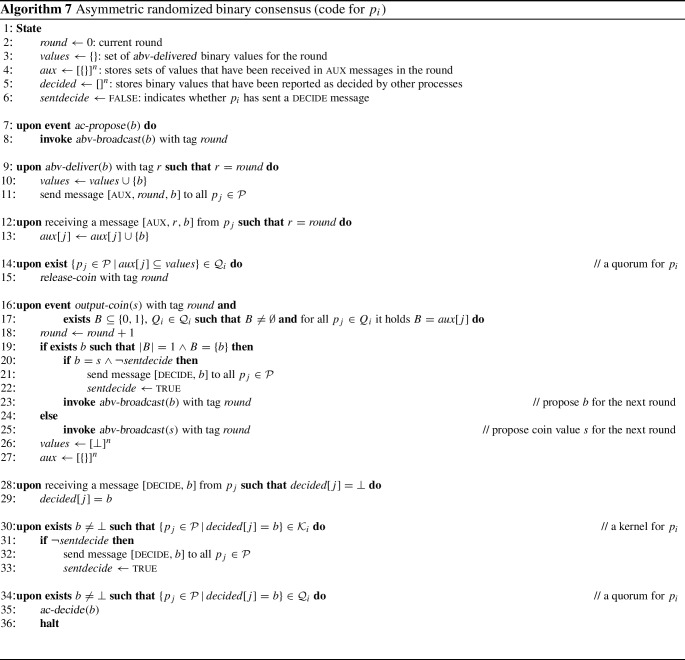


Similar to the protocol of Mostéfaoui et al. [5], Algorithm 7 proceeds in rounds, and in each round an instance of $\textit{abv-broadcast}$ is invoked. A correct process $p_i$ executes $\textit{abv-broadcast}$ and waits for a value *b* identified by a tag characterizing the current round. Once received, $p_i$ adds *b* to $\textit{values}$, broadcasts *b* in an aux message to all other processes, and all of them will eventually add *b* to $\textit{aux}$. The aux messages serve to “enhance” the distributed knowledge about the valid decision values, which must have been *abv-proposed* by processes in the guild. When $p_i$ has received a set $B \subseteq \textit{values}$ of values carried by aux messages from all processes in a quorum $Q_i$ for itself, then $p_i$ releases its coin with tag *r*. Process $p_i$ then waits for $\textit{output-coin}$ with tag *r* and the common coin value *s*. Observe that Algorithm 7 allows the set *B* to change while reconstructing the common coin (Lines 16–17).

Subsequently, $p_i$ checks if there is a single value *b* in *B*. If so, and if $b=s$, then $p_i$ becomes ready to decide *b* and it does so by broadcasting a decide message with value *b* to every process. If there is more than one value in *B*, then $p_i$ changes its proposal to *s*. In any case, the process starts another round and invokes a new instance of $\textit{abv-broadcast}$ with its proposal.

In parallel, the protocol potentially disseminates decide messages and may terminate. When $p_i$ receives a decide message from a kernel of processes for itself containing the same value *b*, then it broadcasts a decide message itself containing *b* to every process, unless it has already done so. Once $p_i$ has received a decide message from a quorum of processes for itself with the same value *b*, it $\textit{ac-decides}(b)$ and halts. This “amplification” step is reminiscent of Bracha’s reliable broadcast protocol [21]. Hence, the protocol does not execute rounds forever, in contrast to the original formulation of Mostéfaoui et al. [5], which satisfies a weaker notion of termination.

The following lemma illustrates that the problem described by Tholoniat and Gramoli [27] and described in Appendix A does not occur in this protocol. This lemma is not directly used in the analysis of Algorithm 7.

#### Lemma 18

If a wise process $p_i$ executes $\textit{output-coin}(s)$ and has $B = \{0,1\}$, then every other wise process that $\textit{output-coin}(s)$ has also $B=\{0,1\}$.

#### Proof

Let us assume that a wise process $p_i$ executes $\textit{output-coin}(s)$ while it stores $B = \{0,1\}$. By inspection of the common-coin implementation, this means that $p_i$ has received share messages from every process in some guild $\mathcal {G}$ (Line 12, Algorithm 5) and has $B=\textit{aux}[j]=\{0,1\}$ for all $p_j$ in a quorum $Q_i$ for $p_i$. Observe that because $p_i$ is wise, $Q_i \cap \mathcal {G}$ contains some correct process.

Consider another wise process $p_j$ that has also obtained $\textit{output-coin}(s)$. It follows that $p_j$ has received share messages from some guild $\mathcal {G}'$ as well. Observe that $p_i$ and $p_j$, before receiving the share messages from every process in $\mathcal {G}$ and $\mathcal {G}'$, respectively, receive all aux messages that the correct processes in these guilds have sent before the share messages. This follows from the assumption of FIFO reliable point-to-point links across the protocols.

Recall from Lemma 10 that the set of guilds is a symmetric Byzantine quorum system for the tolerated system $\mathcal {T}$ of $\mathbb {Q}$. Quorum consistency then implies that $\mathcal {G}$ and $\mathcal {G}'$ have some correct process(es) in common. So, according to the reasoning above, $p_i$ and $p_j$ receive some aux messages from the same correct process before they may output the coin. This means that if $p_i$ has $B=\{0,1\}$ after $\textit{output-coin}(s)$, then every quorum $Q_j$ for $p_j$ will contain a process $p_k$ such that $\textit{aux}[k]=\{0,1\}$ for $p_j$. Every wise process therefore must eventually have $B=\{0,1\}$. $\square$

#### Theorem 19

Algorithm 7 implements asymmetric strong Byzantine consensus.

#### Proof

To prove the *strong validity* property, let us assume that a wise process $p_i$ has $\textit{ac-decided}$ a value *b*. This means that $p_i$ has received $[\textsc {decide}, {b}]$ messages from a quorum $Q_i$ for itself. Moreover, before deciding, process $p_i$ has received $[\textsc {decide}, {b}]$ messages from a kernel $\mathcal {K}_i$ for itself and sent $[\textsc {decide}, {b}]$ to every other process.

Whenever a correct process $p_i$ has sent such a decide message containing *b* in a round *r*, it has obtained $B = \{b\}$ and *b* is the same as the coin value in the round. Then, $p_i$ has received *b* from a quorum $Q_i$ for itself through aux messages. Every process in $Q_i$ has received a $[\textsc {aux}, {r,b}]$ message and *b* has been *abv-delivered*. According to the integrity property of the validated broadcast, *b* has been *abv-broadcast* by a process in the maximal guild and, specifically, $\textit{values}$ contains only values *abv-broadcast* by processes in the maximal guild. It follows that *b* has been proposed by some processes in the maximal guild.

For the *agreement* property, suppose that a wise process has received $[\textsc {aux}, {r,b}]$ messages from a quorum $Q_i$ for itself. Consider any other wise process $p_j$ that has received a quorum $Q_j$ for itself of $[\textsc {aux}, {r,\overline{b}}]$ messages. If at the end of round *r* there is only one value in *B*, then from consistency property of quorum systems, it follows $b = \overline{b}$. Furthermore, if $b=s$ then $p_i$ and $p_j$ broadcast a $[\textsc {decide}, {b}]$ message to every process and decide for *b* after receiving a quorum of $[\textsc {decide}, {b}]$ messages for themselves, otherwise they both $\textit{abv-broadcast}(b)$ and they continue to $\textit{abv-broadcast}(b)$ until $b=s$. If *B* contains more than one value, then $p_i$ and $p_j$ proceed to the next round and invoke a new instance of $\textit{abv-broadcast}$ with *s*. Therefore, at the beginning of the next round, the proposed values of all wise processes are equal. The property easily follows.

For the *integrity* property, notice that the process halts after *ac-deciding* and therefore does not *ac-decide* more than once.

The *probabilistic termination* property follows from two observations. First, the termination and the agreement properties of binary validated broadcast imply that every wise process *abv-delivers* the same binary value from the validated broadcast instance and this value has been *abv-broadcast* by some processes in the maximal guild. Second, we show that with probability 1, there exists a round at the end of which all processes in $\mathcal {G} _{\text {max}}$ have the same proposal *b*. If at the end of round *r*, every process in $\mathcal {G} _{\text {max}}$ has proposed the coin value (Line 25, Algorithm 7), then all of them start the next round with the same value. Similarly, if every process in $\mathcal {G} _{\text {max}}$ has executed Line 23 (Algorithm 7) they adopt the value *b* and start the next round with the same value.

However, it could be the case that some wise process in the maximal guild *abv-broadcasts* a bit *b* in Line 23 and another such process *abv-broadcasts* the coin output *s* in Line 25. Observe that the properties of the common coin abstraction guarantee that the coin value is random and chosen independently of *b*.

In particular, the *unpredictability* of the common coin ensures that no information about *s* is revealed until some kernel *K* for all wise processes, which consists only of correct processes, has released the coin. But for every wise process $p_i$, this kernel *K* will intersect a quorum $Q_i$ in the condition of Line 17. Since the aux messages from the processes $Q_i$ determine $B = \{b\}$ for every wise process that *abv-broadcasts* *b*, all processes in $K \cap Q_i$ must have received the same value *b* before information about the coin can become public. Hence *b* is independent of the random value *s* and they with probability $\frac{1}{2}$. The probability that there exists a round $r'$ in which the coin equals the value *b* proposed by all processes in $\mathcal {G} _{\text {max}}$ during round $r'$ approaches 1 when *r* goes to infinity.

Let *r* thus be some round in which every process in $\mathcal {G} _{\text {max}}$ *abv-broadcasts* the same value *b*; then, none of them will ever change their proposal again. This is due to the fact that every wise process invokes an binary validated broadcast instance with the same proposal *b*. According to the validity and agreement properties of asymmetric binary validated broadcast, every wise process then *bv-delivers* the same, unique value *b*. Hence, the proposal of every wise process is set to *b* and does not change in future rounds. Finally, the properties of common coin guarantee that the processes eventually reach a round in which the coin outputs *b*. Therefore, with probability 1 every process in the maximal guild sends a decide message with value *b* to every process in that round. This implies that it exists a quorum $Q_i \subseteq \mathcal {G} _{\text {max}}$ for a process $p_i \in \mathcal {G} _{\text {max}}$ such that every process in $Q_i$ has sent a decide message with value *b* to every process. Moreover, the set of processes in the maximal guild contains a kernel for $p_i$ and for every other correct process $p_j$ (Corollary 8). If a correct process $p_j$ receives a decide message with value *b* from a kernel for itself, it sends a decide message with value *b* to every process unless it has already done so. It follows that eventually every wise process receives decide messages with the value *b* from a quorum for itself and *ac-decides* for *b*. $\square$

## Conclusion

This work has introduced asymmetric Byzantine quorum systems, which enable distributed fault-tolerant protocols with subjective trust assumptions. The asymmetric-trust model is a strict generalization of Byzantine quorum systems and intended to work with generic extensions of the standard protocols, where Byzantine quorums are used. Indeed, this paper has shown how register emulations, Byzantine consistent and reliable broadcasts, and randomized asynchronous consensus can be extended to asymmetric trust. Some of existing protocols had to be changed in subtle ways because not only asymmetric quorums play a role but also further concepts, such as core sets and kernels. This work has also extended these notions to asymmetric trust.

The changes to existing protocols follow a general pattern. The most important one is that when a process $p_i$ obtains a number of responses from a (Byzantine) quorum, which consists in the threshold case of any set with more than $\lceil \frac{n+f+1}{2} \rceil < n-f$ processes, this is replaced by the step of $p_i$ receiving responses from one of its quorums $Q_i$. Waiting for a core set of responses, which means $f+1$ messages in the threshold case, changes to obtaining a core set $C_i$ for $p_i$ of responses or a kernel $K_i$ for $p_i$ of responses, respectively. The appropriate notion depends on the context.

There exist a considerable number of more elaborate distributed protocols in the Byzantine-fault model, notably for consensus and total-order broadcast. It is expected that these can be generalized as well to asymmetric quorums, but the actual formulations remain open. Furthermore, many Byzantine-tolerant distributed protocols rely on distributed cryptographic primitives. It is an interesting problem to generalize them to subjective trust assumptions in a scalable and efficient way.

## Figures and Tables

**Fig. 7:**
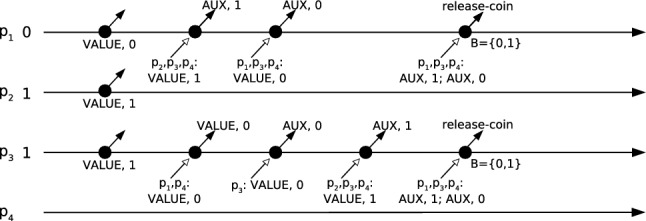
The execution of Algorithm 8, where processes $p_1$ and $p_3$ execute Line 12 with $B=\{0,1\}$

**Fig. 8:**
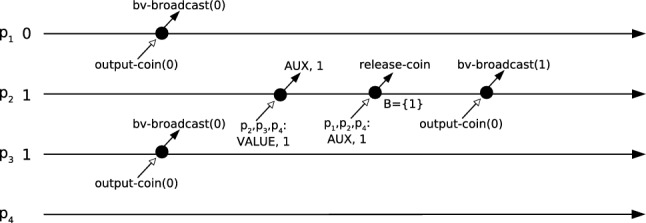
Continuing the execution for the case $s=0$: Process $p_2$ executes Line 12 with $B=\{1\}$. Processes $p_1$ and $p_3$ have already proposed the coin value $s=0$ for the next round, but $p_2$ proposes $\overline{s} = 1$

## Revisiting signature-free asynchronous Byzantine consensus

In 2014, Mostéfaoui et al. [4] introduced a round-based asynchronous randomized consensus algorithm for binary values. It had received considerable attention because it was the first protocol with optimal resilience, tolerating up to $f < \frac{n}{3}$ Byzantine processes, that did not use digital signatures. Hence, this protocol needs only authenticated channels and remains secure against a computationally unbounded adversary. Moreover, it takes $O(n^2)$ constant-sized messages in expectation and has a particularly simple structure. Our description here excludes the necessary cost for implementing randomization, for which the protocol relies on an abstract common coin primitive, as defined by Rabin [24].

This protocol, which we call the *PODC-14* version [4] in the following, suffers from a subtle and little-known problem. It may violate liveness, as has been explicitly mentioned by Tholoniat and Gramoli [27]. The corresponding journal publication by Mostéfaoui et al. [5], to which we refer as the *JACM-15* version, touches briefly on the issue and goes on to present an extended protocol. This fixes the problem, but requires also many more communication steps and adds considerable complexity.

The purpose of this appendix is to revisit the PODC-14 protocol, to point out in detail how the protocol may fail, and to introduce a compact solution for fixing it, all in a self-contained way. For the same reason, we use the symmetric threshold-fault model here, where any *f* out of *n* processes may be faulty.

We discovered discovered this solution while extending the randomized consensus algorithm to asymmetric quorums. The corresponding asymmetric randomized Byzantine consensus protocol appeared in Sect. 7 and is proven secure there.

Before addressing randomized consensus, we recall the key abstraction introduced in the PODC-14 paper, a protocol for broadcasting binary values.

### Binary-value broadcast

The *binary validated broadcast* primitive has been introduced in the PODC-14 version [4] under the name *binary-value broadcast*.^1^ In this primitive, every process may broadcast a bit $b \in \{0,1\}$ by invoking $\textit{bv-broadcast}(b)$. The broadcast primitive outputs at least one value *b* and possibly also both binary values through a $\textit{bv-deliver}(b)$ event, according to the following notion.

#### Definition 14

(*Binary validated broadcast*) A protocol for *binary validated broadcast* satisfies the following properties:

- *Validity:* If at least $(f+1)$ correct processes *bv-broadcast* the same value $b \in \{0,1\}$, then every correct process eventually *bv-delivers* *b*.
- *Integrity:* A correct process *bv-delivers* a particular value *b* at most once and only if *b* has been *bv-broadcast* by some correct process.
- *Agreement:* If a correct process *bv-delivers* some value *b*, then every correct process eventually *bv-delivers* *b*.
- *Termination:* Every correct process eventually *bv-delivers* some value *b*.

The implementation given by Mostéfaoui et al. [4] works as follows. When a correct process $p_i$ invokes $\textit{bv-broadcast}(b)$ for $b \in \{0,1\}$, it sends a value message containing *b* to all processes. Afterwards, whenever a correct process receives value messages containing *b* from at least $f+1$ processes and has not itself sent a value message containing *b*, then it sends such message to every process. Finally, once a correct process receives value messages containing *b* from at least $2f+1$ processes, it delivers *b* through $\textit{bv-deliver}(b)$. Note that a process may *bv-deliver* up to two values. A formal description, in the asymmetric model, appeared in Algorithm 6 (Sect. 7).

### Randomized consensus

We recall the notion of *randomized Byzantine consensus* here and its implementation by Mostéfaoui et al. [4]. In a consensus primitive, every correct process proposes a value *v* by invoking $\textit{propose}(v)$, which typically triggers the start of the protocol among processes; it obtains as output a decided value *v* through a $\textit{decide}(v)$ event. There are no assumptions made about the faulty processes. We use the probabilistic termination property for round-based protocols. It requires that the probability that a correct process decides after executing infinitely many rounds approaches 1.

#### Definition 15

(*Strong Byzantine consensus*) A protocol for asynchronous *strong Byzantine consensus* satisfies:

- *Probabilistic termination:* Every correct process $p_i$ decides with probability 1, in the sense that
  $$\begin{aligned} \lim _{r \rightarrow + \infty } \textrm{P}[\text {a correct process }p_i\text { decides by round }r] = 1. \end{aligned}$$
- *Strong validity:* A correct process only decides a value that has been proposed by some correct process.
- *Integrity:* No correct process decides twice.
- *Agreement:* No two correct processes decide differently.

The probabilistic termination and integrity properties together imply that every correct process decides exactly once, while the agreement property ensures that the decided values are equal. Strong validity asks that if all correct processes propose the same value *v*, then no correct process decides a value different from *v*. Otherwise, a correct process may only decide a value that was proposed by some correct process [28]. In a *binary* consensus protocol, as considered here, only 0 and 1 may be proposed.

The implementation of randomized consensus by Mostéfaoui et al. [4] delegates its probabilistic choices to a *common coin* abstraction [24, 28], a random source observable by all processes but unpredictable for an adversary. A common coin is invoked at every process by triggering a *release-coin* event. We say that a process *releases* a coin because its value is unpredictable, unless more than *f* correct processes have invoked the coin. The value $s \in \mathcal {B}$ of the coin with tag *r* is output through an event *output-coin*.

#### Definition 16

(*Common coin*) A protocol for *common coin* satisfies the following properties:

- *Termination:* Every correct process eventually outputs a coin value.
- *Unpredictability:* Unless more than 2*f* processes have released the coin, no process has any information about the coin output by a correct process.
- *Matching:* With probability 1 every correct process outputs the same coin value.
- *No bias:* The distribution of the coin is uniform over $\mathcal {B}$.

Observe that the unpredictability condition implies that at least $f+1$ *correct* processes are required to release the coin in order for a process to have information about the coin value output by a correct process.

We now recall the implementation of strong Byzantine consensus according to Mostéfaoui et al. [4] in the PODC-14 version, shown in Algorithm 8. A correct process *proposes* a binary value *b* by invoking $\textit{rbc-propose}(b)$; the consensus abstraction *decides* for *b* through an $\textit{rbc-decide}(b)$ event.

The algorithm proceeds in rounds. In each round, an instance of $\textit{bv-broadcast}$ is invoked. A correct process $p_i$ executes $\textit{bv-broadcast}$ and waits for a value *b* to be *bv-delivered*, identified by a tag characterizing the current round. When such a bit *b* is received, $p_i$ adds *b* to $\textit{values}$ and broadcasts *b* through an aux message to all processes. Whenever a process receives an aux message containing *b* from $p_j$, it stores *b* in a local set $\textit{aux}[j]$. Once $p_i$ has received a set $B \subseteq \textit{values}$ of values such that every $b \in B$ has been delivered in aux messages from at least $n-f$ processes, then $p_i$ releases the coin for the round. Subsequently, the process waits for the coin protocol to output a binary value *s* through $\textit{output-coin}(s)$, tagged with the current round number.

Process $p_i$ then checks if there is a single value *b* in *B*. If so, and if $b=s$, then it decides for value *b*. The process then proceeds to the next round with proposal *b*. If there is more than one value in *B*, then $p_i$ changes its proposal to *s*. In any case, the process starts another round and invokes a new instance of $\textit{bv-broadcast}$ with its proposal. Note that the protocol appears to execute rounds forever.

### A liveness problem

Tholoniat and Gramoli [27] mention a liveness issue with the randomized algorithm in the PODC-14 version [4], as presented in the previous section. They sketch a problem that may prevent progress by the correct processes when the messages between them are received in a specific order. In the JACM-15 version, Mostéfaoui et al. [5] appear to be aware of the issue and present a different, more complex consensus protocol.

We give a detailed description of the problem in Algorithm 8. Recall the implementation of binary-value broadcast, which disseminates bits in value messages. According to our model, the processes communicate by exchanging messages through an asynchronous reliable point-to-point network. Messages may be reordered, as in the PODC-14 version.

Let us consider a system with $n = 4$ processes and $f = 1$ Byzantine process. Let $p_1, p_2$ and $p_3$ be correct processes with input values 0, 1, 1, respectively, and let $p_4$ be a Byzantine process with control over the network. Process $p_4$ aims to cause $p_1$ and $p_3$ to release the coin with $B = \{0,1\}$, so that they subsequently propose the coin value for the next round. If messages are scheduled depending on knowledge of the round’s coin value *s*, it is possible, then, that $p_2$ releases the coin with $B = \{\overline{s}\}$. Subsequently, $p_2$ proposes also $\overline{s}$ for the next round, and this may continue forever. We now work out the details, as illustrated in Figs. 7 and 8.

First, $p_4$ may cause $p_1$ to receive $2f+1$ $[\textsc {value},1]$ messages, from $p_2, p_3$ and $p_4$, and to $\textit{bv-deliver}$ 1 sent at the start of the round. Then, $p_4$ sends $[\textsc {value}, 0]$ to $p_3$, so that $p_3$ receives value 0 twice (from $p_1$ and $p_4$) and also broadcasts a $[\textsc {value},0]$ message itself. Process $p_4$ also sends 0 to $p_1$, hence, $p_1$ receives 0 from $p_3$, $p_4$, and itself and therefore $\textit{bv-delivers}$ 0. Furthermore, $p_4$ causes $p_3$ to $\textit{bv-deliver}$ 0 by making it receive $[\textsc {value},0]$ messages from $p_1$, $p_4$, and itself. Hence, $p_3$ $\textit{bv-delivers}$ 0. Finally, process $p_3$ receives three $[\textsc {value}, 1]$ messages (from itself, $p_2$, and $p_4$) and $\textit{bv-delivers}$ also 1.

Recall that a process may broadcast more than one $\textsc {aux}$ message. In particular, it broadcasts an $\textsc {aux}$ message containing a bit *b* whenever it has bv-delivered *b*. Thus, $p_1$ broadcasts first $[\textsc {aux}, 1]$ and subsequently $[\textsc {aux}, 0]$, whereas $p_3$ first broadcasts $[\textsc {aux}, 0]$ and then $[\textsc {aux}, 1]$. Process $p_4$ then sends to $p_1$ and $p_3$ $\textsc {aux}$ messages containing 1 and 0. After delivering all six aux messages, both $p_1$ and $p_3$ finally obtain $B = \{0,1\}$ in Line 12 (Algorithm 8) and see that $|B| \ne 1$ in L 16 (Algorithm 8). Processes $p_1$, $p_3$ and $p_4$ invoke the common coin.

The Byzantine process $p_4$ may learn the coin value as soon as $p_1$ or $p_3$ have released the common coin, according to unpredictability. Let *s* be the coin output. We distinguish two cases:

*Case*$s = 0$: Process $p_2$ receives now three $[\textsc {value}, 1]$ messages, from $p_3$, $p_4$ and itself, as shown in Fig. 8. It $\textit{bv-delivers}$ 1 and broadcasts an $[\textsc {aux}, 1]$ message. Subsequently, $p_2$ delivers three $\textsc {aux}$ messages containing 1, from $p_1, p_4$ and itself, but no $[\textsc {aux}, 0]$ message. It follows that $p_2$ obtains $B = \{1\}$ and proposes 1 for the next round in Line 19. On the other hand, $p_1$ and $p_3$ adopt 0 as their new proposal for the next round, according to Line 21. This means that no progress was made within this round. The three correct processes start the next round again with differing values, again two of them propose one bit and the remaining one proposes the opposite.
*Case*$s = 1$: Process $p_4$ sends $[\textsc {value}, 0]$ to $p_2$, so that it delivers two value messages containing 0 (from $p_1$ and $p_4$) and thus also broadcast $[\textsc {value}, 0]$ (this execution is not shown). Recall that $p_3$ has already sent $[\textsc {value}, 0]$ before. Thus, $p_2$ receives $n-f$ $[\textsc {value}, 0]$ messages, $\textit{bv-delivers}$ 0, and also broadcasts an $\textsc {aux}$ message containing 0. Subsequently, $p_2$ may receive $n-f$ messages $[\textsc {aux}, 0]$, from $p_3$, $p_4$, and itself. It follows that $p_2$ executes Line 12 with $B = \{0\}$ and chooses 0 as its proposal for the next round (in Line 19). On the other hand, also here, $p_1$ and $p_3$ adopt the coin value $s=1$ and propose 1 for the next round in Line 21. Hence, no progress has been made in this round, as the three correct processes enter the next round with differing values.

The protocol may continue like this forever, producing an infinite execution with no termination.

### Fixing the problem

We show how the problem can be prevented with a conceptual insight and two small changes to the original protocol. We do this by recalling the example just presented. The complete protocol and a formal proof are given in Sect. 7, using the more general model of asymmetric quorums.

We start by considering the nature of the common coin abstraction: In any full implementation, the coin is not an abstract oracle, but implemented by a concrete protocol that exchanges messages among the processes.

Observe now that in the problematic execution, the network reorders messages between correct processes. Our first change, therefore, is to assume FIFO ordering on the reliable point-to-point links. This may be implemented over authenticated links, by adding sequence numbers to messages and maintaining a buffer at the receiver [28]. Consider $p_2$ in the example and the messages it receives from the other correct processes, $p_1$ and $p_3$. W.l.o.g. any protocol implementing a common coin requires an additional message exchange, where a correct process sends at least one message to every other process, say, a coin message with arbitrary content (to be specific, see Algorithm 5, Sect. 7). Observe that at least $f+1$ *correct* processes are required to send a coin message in order for a process to have information about the coin value.

When $p_2$ waits for the output of the coin, it needs to receive, again w.l.o.g., a coin message from $n-f$ processes. Since the other two correct processes ($p_1$ and $p_3$) have sent two value messages and aux messages each before releasing the coin, then $p_2$ receives these messages from at least one of them before receiving enough coin messages, according to the overlap among Byzantine quorums.

This means that $p_2$ cannot satisfy the condition in Line 12 with $|B| = 1$. Thus the adversary may no longer exploit its knowledge of the coin value to prevent termination. (Mostéfaoui et al. [5] (JACM-15) remark in retrospect about the PODC-14 version that a “fair scheduler” is needed. However, this comes without any proof and thus remains open, especially because the JACM-15 version introduces a much more complex version of the protocol.)

Our second change is to allow the set *B* to dynamically change while the coin protocol executes. In this way, process $p_2$ may find a suitable *B* according to the received aux messages while concurrently running the coin protocol. Eventually, $p_2$ will have output the coin *and* its set *B* will contain the same values as the sets *B* of $p_1$ and $p_3$. Observe that this dynamicity is necessary; process $p_2$ could start to release the coin after receiving $n-f$ aux messages containing only the value 1. However, following our example, due to the assumed FIFO order, it will receive from another correct process also an $\textsc {aux}$ message containing the value 0, before the coin message. If we do not ask for the dynamicity of the set *B*, process $p_2$, after outputting the coin, will still have $|B|=1$. Mostéfaoui et al. in the PODC-14 version ([4, Fig. 2, Line 5]) seem to rule this out.

Observe that the common-coin primitive here requires *more than f correct* processes to release the coin before it may be predicted by the faulty processes. Within an implementation, this translates into receiving a coin message from more than 2*f* processes (or $2f+1 = n-f$ processes, in case $n = 3f+1$). Abraham et al. [46, 47] show that such an assumption (which they call an 2*f*-unpredictable coin) is necessary in order to prevent this liveness problem. With an ordinary coin primitive (i.e., one where at least *one correct process* is required to send a coin message, before information about the coin value may become available), an adversary would still be able to produce an infinite execution and to violate termination [47, Appendix A].

## Declarations

### Publication history

This work combines and subsumes multiple preliminary publications on asymmetric trust and protocols with asymmetric trust, which appeared at OPODIS 2019 [48], DISC 2021 [49], and ESORICS-CBT 2021 [50].
